# Phytosulfokine alpha enhances regeneration of transformed and untransformed protoplasts of *Brassica oleracea*

**DOI:** 10.3389/fpls.2024.1379618

**Published:** 2024-03-27

**Authors:** Valentin Vogrinčič, Damijana Kastelec, Jana Murovec

**Affiliations:** Biotechnical Faculty, University of Ljubljana, Ljubljana, Slovenia

**Keywords:** PSK-α, cell division, differentiation, organogenesis, alginate disc, genetic transformation, plant genome editing, plasmid

## Abstract

Phytosulfokine-α (PSK-α) is a disulfated pentapeptide (YIYTQ) acting as an intercellular signal peptide and growth factor. It was originally isolated from conditioned medium of asparagus mesophyll cell cultures in 1996 and later characterized as a hormone-like signal molecule with important roles in numerous processes of *in vivo* plant growth and development. It is currently becoming a valuable mitogenic factor in plant breeding and biotechnology due to its stimulatory effect on *in vitro* cell elongation, proliferation and differentiation. The focus of our work was to review current knowledge about the roles of PSK-α in plant biotechnology and to evaluate its influence on the regeneration of protoplasts of four *Brassica oleracea* cultivars (two cauliflower and two cabbage) cultured under two distinctive protocols and with different protoplast densities. Protoplast regeneration was studied due to its high value for plant genome editing, which is generally limited by the inefficient regeneration of treated protoplasts of numerous important plant genotypes. Our hypothesis was that the stress related to PEG-mediated protoplast transformation and the following decrease in viable protoplast density in culture could be alleviated by the addition of PSK-α to the culture medium. We therefore tested whether PSK-α could increase cell division at the early stages of culture (5 and 15 days after protoplast isolation) and stimulate the formation of microcallus colonies up to the 30st day of culture and to evaluate its influence on callus organogenesis leading to shoot regeneration. The PSK-α showed a strong stimulatory effect on untransformed protoplast regeneration already during the first days of culture, accelerating cell division up to 5.3-fold and the formation of multicellular microcallus colonies up to 37.0-fold. The beneficial influence was retained at later stages of regeneration, when PSK improved shoot organogenesis even if it was present only during the first 10 days of culture. The highest numbers of shoots, however, were regenerated when PSK was present during the first days of culture and later in solid shoot regeneration medium. Finally, the addition of PSK-α to PEG-transformed protoplasts significantly enhanced their division rate and the formation of microcallus colonies in selection media, up to 44.0-fold.

## Highlights

- Phytosulfokine alpha (PSK-α) improves protoplast regeneration by accelerating early-stage cell division and differentiation of callus cells into shoots. It compensates for the drop of viable protoplast density after PEG-mediated transformation.

## 1 Introduction

Cells in a multicellular plant organism employ various mechanisms for intercellular communication to establish the necessary cell identity, activity and patterning and to regulate cell division, growth, structure and functions of plant tissues and organs. They use plant growth regulators (phytohormones), mobile transcription factors, non-coding small RNAs (microRNAs), and small signal peptides secreted in the apoplast, and non-secreted peptides perform their functions in the cytoplasm (Van Norman et al., 2011). Biochemical, genetic and bioinformatics studies in the last decades have discovered extracellular and intracellular peptides that are indispensable components of intercellular communication. These peptides define and coordinate a myriad of cellular functions in the plant organism and are involved in plant growth and development, in defense responses or in symbiosis. In the last decades, the number of annotated and characterized plant signal peptides has increased multiple folds and now vastly exceeds that of well-known plant hormones (Matsubayashi and Sakagami, 2006; Ohyama et al., 2008; Matsubayashi, 2014). Most of these peptides were identified in bioassay-based biochemical and forward genetic studies. *In silico* genomic analysis has also identified the presence of a multitude of uncharacterized genes that contain short open reading frames homologous to those of known peptides (Lease and Walker, 2006).

The group of peptide signals is extensive and contains some prominent members and founders of peptide families, such as systemin and systemin-like peptides, identified as components of the systemic wound response in the family Solanaceae (Pearce et al., 1991; Constabel et al., 1998), CLAVATA 3 peptide, and other compounds of the CLE peptide family discovered in *Arabidopsis thaliana* and involved in the organization of the shoot apical meristem (Fletcher et al., 1999). Further, the *ENOD40* gene in rice and leguminous species encodes small (10–13 amino acids) peptides involved in the initiation of cortical cell division upon infection with *Rhizobia* (van de Sande et al., 1996). The S-Locus Cysteine Rich Protein (SCR or SP11) plays a role in the self-incompatibility mechanism in the family Brassiceace (Vanoosthuyse et al., 2001), and, phytosulfokines (PSKs) were first detected in proliferating suspension cultures of *Asparagus officinalis* mesophyll cells (Matsubayashi and Sakagami, 1996; Matsubayashi et al., 1996; Ohyama et al., 2008). Matsubayashi and Sakagami, as well as numerous other researchers before them, noticed that the addition of filtered medium from freshly established or highly proliferating suspension cultures (i.e., conditioned medium) of homologous or heterologous species to suspension cultures leads to increased proliferation. Biochemical mass spectrometric analysis of chromatography fractions from the conditioned medium led Matsubayashi and Sakagami to the identification of two sulfated oligopeptides, PSK-α and PSK-β. Of these, PSK- α is the main active form of PSK and as such used in plant biotechnology, whereas PSK-β is an enzymatically truncated form of PSK-α with a 10 times lower biological activity. Phytosulfokines are commonly abbreviated by the term PSK, which almost always refers to PSK-α, whose biological activity is dependent on tyrosine sulfation and proteolytic processing (Matsubayashi and Sakagami, 1996; Matsubayashi et al., 1996). Later, PSKs have been later identified in conditioned medium of rice (Matsubayashi et al., 1997) and carrot (Hanai et al., 2000), which reflects their broader presence in the plant kingdom. Although PSK were first detected as secreted peptides in conditioned medium, expression studies have indicated that PSK molecules are also produced in intact plants (Yang et al., 1999; Lorbiecke and Sauter, 2002).

There are several prominent and well reported roles of PSKs both *in vivo* and *in vitro*, such as the promotion of cell expansion and division (Yu et al., 2016, 2019), the attenuation of stress responses to biotic (Mosher et al., 2013; Yang et al., 2019) and abiotic factors (Song et al., 2017; Liu et al., 2021; Stührwohldt et al., 2021), the promotion of cell longevity, delaying organ senescence, primary root growth and adventitious root formation (Wu et al., 2019; Kaufmann et al., 2021), pollen tube growth, male sterility (Yu et al., 2016), and a role in organogenesis, plant sexual reproduction, and even in cotton fiber cell elongation *in vitro* (Han et al., 2014). The PSK signaling in *Arabidopsis* explants cultured *in vitro* accelerates cell division, which results in the formation of larger calli (Matsubayashi et al., 2006). They also stimulate somatic embryogenesis in carrot cell suspensions with low embryogenic competence by stimulating cell division frequency in developing embryos (Kobayashi et al., 1999; Hanai et al., 2000). Their positive effect on the induction of somatic embryogenesis has also been demonstrated in gymnosperms, such as sugi (*Cryptomeria japonica* D.Don) (Igasaki et al., 2003), Japanese larch (*Larix leptolepis* Gordon) (Umehara et al., 2005), and slash pine (*Pinus elliottii* Engelm.) (Yang et al., 2020). Moreover PSK also stimulate microspore embryogenesis for the regeneration of (doubled) haploid plants of *Brassica napus* (Mestinšek Mubi et al., 2024), wheat, and triticale (Asif et al., 2014).

So far, the effect of PSK on *in vitro* protoplast proliferation has been evaluated in two culture systems: protoplasts in suspensions and protoplasts embedded in alginate or agarose discs. Matsubayashi and Sakagami (1996) were the first to induce the proliferation of dispersed *Asparagus* mesophyll protoplasts in suspension at a density too low for successful protoplast division (4 x 10^4^ protoplasts mL^-1^) by adding synthetic PSK at concentrations from 10^-9^ to 10^-7^ M. Later, these authors showed a favorable mitogenic effect of PSK at low protoplast density also on rice protoplasts embedded in agarose discs (Matsubayashi et al., 1997). In contrast to suspension culture, embedding cells in a thin layer of support matrix maximizes the distances between them and, thus, minimizes the effective concentrations of growth factors produced by the cells themselves. This enables an unbiased evaluation of the effect of added PSK (or other mitogenic factors such as hormones) in culture medium on protoplast regeneration.

In the past years, protoplasts have gained great importance in plant biotechnology since their transient transformation with genome editing vectors can circumvent transgenesis for the creation of improved cultivars. However, as comprehensively addressed in a recent publication of Reed and Bargmann (2021), the recovery of successfully edited plants presents a significant bottleneck in the application of new plant breeding technologies, and the methods vary substantially depending on the species’ genotype (Reed and Bargmann, 2021). Our aim in this study was therefore first to evaluate the regeneration success of *Brassica oleracea* protoplasts embedded in alginate discs at densities below the optimal range of 1 x 10^5^ to 1 x 10^6^ protoplasts mL^-1^ (Davey et al., 2010) by using two different protocols for *Brassica oleracea* protoplast culture. Following that, an experiment was performed testing the effect of 0.1 µM PSK-α in culture medium to protoplasts embedded in alginate discs at different densities and evaluating again the percentages of dividing cells, the number of formed microcalli, and the number of regenerated shoots. Finally, we evaluated the effect of PSK on cell division of protoplasts transformed with a plasmid coding for the genome editing of the endogenous *FRIGIDA* gene to verify whether the addition of PSK can compensate for the decrease in viable protoplast density after PEG-mediated transformation.

## 2 Materials and methods

### 2.1 Experimental design

**Figure 1:**
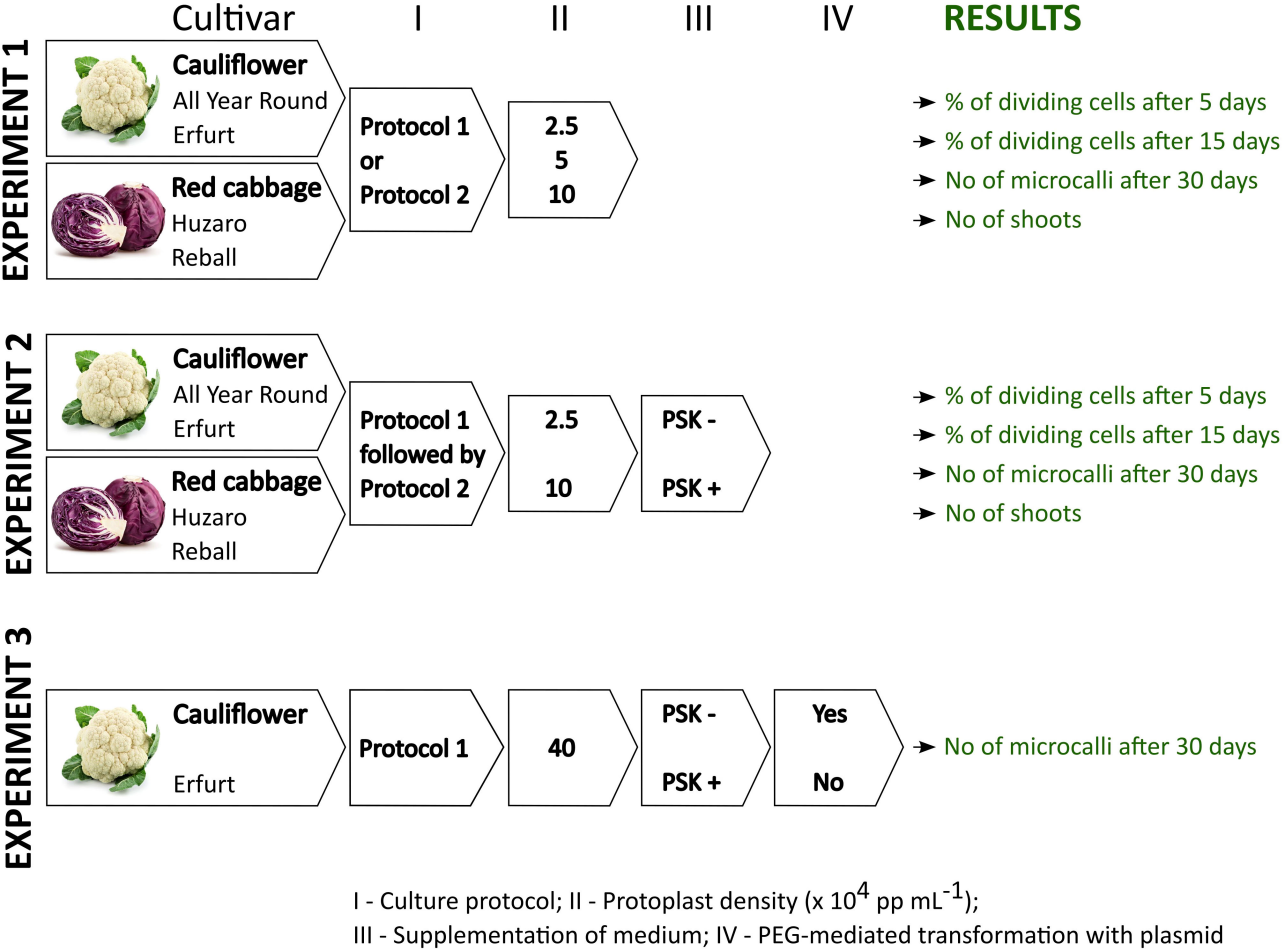
Schematic representation of the experiments and data collection within this study.

The study consisted of three independent experiments of protoplast isolation, culture, and transformation, each of them repeated in three biological replicates at three different dates. The experiments are outlined in **Figure 1** and briefly described below, whereas the detailed protocols are described in the following sections. Throughout the article, the expression ‘protoplast’ is used only for cells without cell walls from the day of protoplasts isolation until re-synthesis of the cell wall. Later, the terms ‘cell’ and ‘multicellular structures’ are used when referring to cell division since protoplasts can only divide after reformation of the cell wall, i.e., once they regain cell characteristics.

**Experiment 1** aimed at elucidating the effect of protoplast density and the culture protocol on cell division at early stages of culture, microcallus formation and shoot regeneration of four *Brassica oleracea* cultivars: All Year Round, Erfurt, Huzaro, and Reball. Two morphotypes of *Brassica oleracea*, cauliflower and cabbage, each with two distinctive cultivars, were included in our study to assess the responses of the four different genotypes to the analyzed parameters. The protoplasts were immobilized in alginate discs at densities of 2.5, 5, or 10 x 10^4^ protoplasts per mL (further referred to as ‘pp mL^-1^’) and cultured following Protocol 1 or Protocol 2. For each treatment, five alginate discs were prepared in the first and second biological replicate and four discs in the third biological replicate. The results were recorded as the number of dividing cells among 150 analysed cells per disc after 5 and after 15 days of culture. The numbers were recalculated to the percentage of dividing cells per disc (i.e., per technical replicate) and later to the percentage of dividing cells per biological replicate. The multicellular structures were further categorized based on the number of cells they contained into categories (2–4 cells, 4–8 cells, more than 8 cells) after 5 and after 15 days of culture. Thirty days after isolation, the number of microcallus colonies (i.e., microcalli) larger than 0.5 mm was determined per disc and normalized to the number of microcalli per 10,000 cultured protoplasts. For each treatment, the mean percentage of dividing cells and the number of microcalli per 10,000 protoplasts were calculated based on the results obtained in 5 (in the 1^st^ and 2^nd^ biological replicate) or 4 (in the 3^rd^ biological replicate) discs. Overall, within Experiment 1, a total of 336 discs were prepared, cultured, and analyzed at three time points. At the end of Experiment 1, organogenesis was evaluated for each treatment and presented as the mean number of regenerated shoots.

**Experiment 2** was focused on the evaluation of the effect of 0.1 µM PSK supplementation in liquid medium for the first 10 days of culture. It was recorded as the percentage of dividing cells after 5 and after 15 days of culture and as the number of formed microcallus colonies after 30 days of culture, as described for the first experiment. The experiment was performed on protoplasts of the same four cultivars, All Year Round, Erfurt, Huzaro, and Reball, which were immobilized at densities of 2.5 and 10 x 10^4^ pp mL^-1^ and cultured following Protocol 1 until day 30, when the formed microcalli were subcultured to solid regeneration medium as described in Protocol 2. Eight discs were prepared for each biological replicate, and the results were collected as the percentages of dividing cells and numbers of microcallus colonies per 10,000 protoplasts. Overall, within Experiment 2, a total of 384 discs were prepared, cultured, and analyzed at three time points. At the end of Experiment 2, organogenesis was evaluated for each treatment and presented as the mean number of regenerated shoots.

**Experiment 3** was designed to evaluate whether supplementation of 0.1 µM PSK in culture medium can promote cell division of transformed protoplasts. To this end, the experiment involved culturing transformed and untransformed protoplasts at an initial immobilization density of 40 x 10^4^ pp mL^-1^ under Protocol 1, with or without the addition 0.1 µM PSK. Data were recorded as the viability of transformed and untransformed protoplasts 2 days after protoplasts isolation and PEG-mediated transformation. Their ability to form microcallus colonies was evaluated 30 days after protoplast isolation and the start of culture. The experiment was performed on protoplasts of the cauliflower cultivar Erfurt, from which three discs were prepared for each treatment and each of the three biological replicates. The results were expressed as the percentages of viable protoplasts 2 days after protoplast isolation and as the mean number of microcallus colonies per alginate disc after 30 days of culture. Overall, within Experiment 3, a total of 216 discs were prepared, cultured, and analyzed.

### 2.2 Plant material

Seeds of the cauliflower (*Brassica oleracea* var. *botrytis*) cultivars All Year Round (Moles seeds, UK) and Erfurt (Semenarna Ljubljana, SI) and the red cabbage (*Brassica oleracea* var. *capitata* f. *rubra*) cultivars Huzaro F1 (Bejo, NL) and Reball F1 (Syngenta, CH) were surface-sterilized in 1.66% sodium dichloroisocyanuric acid, rinsed three times for 5 min with sterile distilled water, and air-dried. Subsequently, the seeds were inoculated in sterile box culture vessels (OS140BOX, Duchefa) containing 50 mL of MS (Murashige and Skoog, 1962) basal medium with 20 g L^-1^sucrose and 1% Daishin agar (Duchefa). Plants were grown in a growth chamber at a temperature of 25 ± 2°C with a 16-hour photoperiod and a light intensity of 55 μ mol m^-2^s^-1^.

### 2.3 Protoplast isolation and culture

Protoplasts were isolated from leaves of 5-week-old *in vitro-*grown plants. Leaves were cut into approximately 2-mm pieces and subjected to plasmolysis in 54.6 g L^-1^ sorbitol, 7.4 g L^-1^ CaCl_2_ x 2 H_2_O, pH 5.8 for 1 h at 25°C. The plasmolysis solution was then removed, and the tissue was treated with an enzyme solution consisting of 0.5% (w/v) cellulase Onozuka R-10 (Duchefa Biocehmie), 0.1% (w/v) macerozyme (Duchefa) in B-medium (Pelletier et al., 1983), pH 5.8, filter-sterilized (0.22 µM syringe filter, TPP). The Petri dishes (90 mm) were placed on a gyratory shaker (30 rpm) and incubated in the enzyme solution for 16 h at 25°C in the dark. After incubation, the solution was filtered through a nylon sieve (40-μm cell strainer, Corning) and centrifuged at 150 x g for 5 minutes in 10-mL round-bottom test tubes (Kartell, IT). The pellets were resuspended in 0.5 M sucrose with 1 mM 2-(N-morpholino)ethanesulfonic acid (MES), pH 5.8. This suspension was overlaid with 2 mL of W5 solution (154 mM NaCl, 125 mM CaCl_2_, 5 mM KCl, 5 mM glucose, pH 5.8) and centrifuged at 190 x g for 10 min. The protoplasts gathered in the interphase between sucrose with MES and W5 solutions were carefully collected, resuspended in W5 solution, and centrifuged again at 150 x g for 5 min. The pellet was resuspended according to the culture protocol, as follows: (1) Protocol 1: B basal medium according to Pelletier and colleagues (Pelletier et al., 1983) or (2) Protocol 2: CPP basal medium consisting of macroelements, microelements, and organic acids, according to Kao and Michayluk (1975), vitamins according to B5 medium (Gamborg et al., 1968), 250 mg/L casein hydrolysate, 74 g/L glucose, 0.1 mg/L 2,4-dichlorophenoxyacetic acid (2,4-D), and 0.2 mg/L zeatin, pH 5.6. The yield of viable protoplasts was determined by staining with fluorescein diacetate (FDA) at a final concentration of 1 µg mL^-1^ and counting the protoplasts in a Neubauer counting chamber. The density of viable protoplasts was adjusted to two times the required density, using the appropriate culture medium. The protoplasts were then immobilized in calcium alginate discs by mixing the protoplast suspension with an equal volume of alginate solution (2.8% alginic acid sodium salt (Sigma-Aldrich), 0.4 M mannitol, pH 5.8, filter-sterilized) to obtain the desired density. Depending on the experiment, the protoplasts were immobilized at different densities: 40 x 10^4^ pp mL^-1^, 10 x 10^4^ pp mL^-1^, 5 x 10^4^ pp mL^-1^, and 2.5 x 10^4^ pp mL^-1^. Subsequently, 100 µL-aliquots of the protoplast-alginate mixtures s were dropped onto 90-mm Petri dishes containing calcium-agar medium (40 mM CaCl_2,_ 0.4 M mannitol, 1% agar (Duchefa), pH 5.8, autoclaved) and spread by slowly rotating the Petri dish. After incubation for 1 hour at room temperature, the polymerized alginate discs with the embedded protoplasts were transferred into 6-well plates (Greiner, Cellstar) containing 2.5 mL of culture medium (B or CPP medium for Protocol 1 or Protocol 2, respectively). Within Experiments 2 and 3, some culture media were supplemented with 0.1 µM PSK-α (PeptaNova, GmbH, DE) for the first 10 days of protoplast culture. Protoplasts in alginate discs were incubated in the dark at 25 ± 2°C. According to culture Protocol 1, the B medium was replaced by C medium after 10 days, which was replaced by D medium after another 10 days. Based on a previous study (Nugent et al., 2006), two modifications were made to the media, as described elsewhere (Pelletier et al., 1983): 0.1 g L^-1^ MES was added to liquid media B, C, and D, and Tween 80 was omitted from medium B. The protoplasts were cultured according to Protocol 2, with fresh CPP medium every 10 days. After 20 days of culture, when the microcalli were macroscopically visible, the cultures were incubated under illumination, with the first 3 days under dim light, followed by a light intensity of 55 μmol m^-2^s^-1^ and a 16-h photoperiod.

### 2.4 Evaluation of callus formation and shoot differentiation

Early cell divisions were counted on days 5 and 15 after protoplast isolation, using the ZOE Flourescent Cell Imager (Bio-Rad). The numbers of dividing cells among 150 cells in three random fields of view within each disc were determined, and the multicellular structures were categorized as containing 2–4 cells, 4–8 cells, and 8 or more cells. After 30 days of culture, the numbers of microcallus colonies larger than 0.5 mm in each alginate disc were determined using a stereomicroscope (Nikon SMZ-745). In the first and second experiments, the numbers of counted microcallus colonies per alginate disc were normalized to the numbers of microcalli per 10,000 cultured protoplasts.

After counting the microcallus colonies at least 0.5 mm in size, the whole alginate discs were transferred onto solid shoot regeneration media according to the culture protocols used. Within Protocol 1, the discs were transferred to solid medium E for callus growth and shoot regeneration and subcultured every 2 weeks to fresh medium E. Protocol 2 involved placing whole alginate discs onto filter papers moistened with a medium that had almost the same composition as CPP, except that the concentration of organic acids was halved; it contained 30 g L^-1^ sucrose instead of glucose, 20 g L^-1^ mannitol was added, and 0.1 mg L^-1^ NAA was replaced with 0.1 mg L^-1^ 2,4-D and was therefore labelled as CPPD (according to Dirks et al., 1996). The filter paper with alginate discs was placed onto solid regeneration medium composed of full-strength Murashige and Skoog (MS) basal medium with vitamins (Duchefa), 20 g L^-1^ sucrose, and 1 mg L^-1^ benzylaminopurine (BAP) (Kiełkowska and Adamus, 2017). The filter paper was removed after 2 weeks, and the calli were transferred to fresh shoot regeneration medium every 2 weeks.

### 2.5 Protoplast transformation

Protoplasts of the cauliflower cultivar Erfurt were transformed with a plasmid (14,155 bp) containing the *GFP* reporter gene under the control of the CaMV 35S promoter and the selectable gene neomycin phosphotransferase (*nptII*) under the control of the *Pnos* promoter, the Cas9 gene under the CaMV 35S promoter, and sgRNA-FRI1 targeting the same locus, as described elsewhere (Murovec et al., 2018).

Protoplasts were resuspended in MMG transformation buffer (4 mM MES, 0.4 M mannitol, 15 mM MgCl_2_, pH 5.7, filter-sterilized) at a density of 2.5 x 10^6^ pp mL^-1^. Each transformation was carried out in a volume of 200 μL in 10-mL centrifuge tubes. For the transformation of approximately 500,000 protoplasts, 20 µL of plasmid DNA was added to the protoplasts. An equal volume of 40% (w/v) polyethylene glycol (PEG4000) was added, and the solution was gently mixed and incubated for 15 minutes in the dark at room temperature. After incubation, an equal volume of W5 was added twice to terminate the reaction. Protoplasts were sedimented with centrifugation, rinsed with culture medium, resuspended at a density of 8 x 10^5^ treated pp mL^-1^, and embedded in 100-µL alginate discs at an immobilization density of 40 x 10^4^ pp mL^-1^. Transformed protoplasts in discs were cultured according to Protocol 1, half of them with the addition of 0.1 µM PSK in the first 10 days of culture. Kanamycin (Duchefa) was added to fresh liquid culture medium 10 days after transformation and throughout the following subcultures at concentrations of 10, 50, or 100 mg L^-1^. As a control, untreated protoplasts were also immobilized at a density of 40 x 10^4^ pp mL^-1^, and the discs were first cultured for 10 days in liquid medium with or without supplementation of 0.1 µM PSK and later on in liquid medium with 10, 50, or 100 mg L^-1^ kanamycin.

The transient expression of the *GFP* gene was evaluated 2 days after transformation by determining the number of fluorescing protoplasts, using the ZOE Flourescent Cell Imager. After that, the protoplasts in alginate discs were stained with FDA, and the percentages of viable protoplasts were calculated based on the numbers of fluorescing and non-fluorescing protoplasts. After 30 days of culture, the number of microcallus colonies per alginate disc was determined.

### 2.6 Statistical analysis

The number of dividing cells after 15 days of culture in Experiments 1 and 2 and the number of microcalli after 30 days of culture in all three experiments were analysed based on a generalized linear model (GLM), assuming different probability distributions of the dependent variables: Poisson, quasi-Poisson, and negative binomial distributions. The latter two were used in cases where model diagnostics revealed excessive dispersion of the residuals. In the case of Experiment 1, cultivar (4 levels: All Year Round, Erfurt, Huzaro, Reball), protocol (two levels: Protocol 1, Protocol 2), and protoplast density (three levels: 2.5, 5, and 10 x 10^4^ pp mL^-1^) with three two-factor interactions (cultivar: protocol, cultivar: density, protocol: density) and one three-factor interaction (cultivar: protocol: density) were included as fixed effects in the model. Mean fold change in the percentage of dividing cells or number of microcalli between treatments was assessed based on contrast analysis controlling for family-specific test error. In Experiment 2, data were analysed as described for Experiment 2, with factor protocol modified by factor supplementation with two levels (PSK+, PSK-). In Experiment 3, only one cultivar was analysed considering three factors: transformation with two levels (untransformed, transformed), supplementation with two levels (PSK+, PSK-), and kanamycin with three levels (10, 50, and 100 mg L^-1^). All statistical analyses were performed using the program R (R Core Team, 2022).

## 3 Results

### 3.1 Protoplast yield and viability

Protoplasts were isolated from all tested cultivars with a yield and viability that allowed successful culture establishment. Protoplast viability after isolation exceeded 90% in all cultivars: All Year Round 92.7 ± 1.7%, Erfurt 93.3 ± 1.6%, Huzaro 93.6 ± 1.3%, and Reball 94.2 ± 1.2%. Protoplast yield, expressed as the number of viable protoplasts per gram of leaf tissue, was also high for all cultivars: All Year Round 9 ± 0.8 x 10^6^ pp g^-1^ of tissue, Erfurt 6.3 ± 0.7 x 10^6^ pp g^-1^ of tissue, Huzaro 5.7 ± 0.8 x 10^6^ pp g^-1^ of tissue, and Reball 7.8 ± 0.6 x 10^6^ pp g^-1^ of tissue. It was therefore concluded that the combination of 0.5% cellulase and 0.1% macerozyme allows high yields of viable protoplasts after overnight digestion of cauliflower and cabbage (*Brassica oleracea*) leaves cultured *in vitro*.

### 3.2 Experiment 1: Cell division

**Figure 2:**
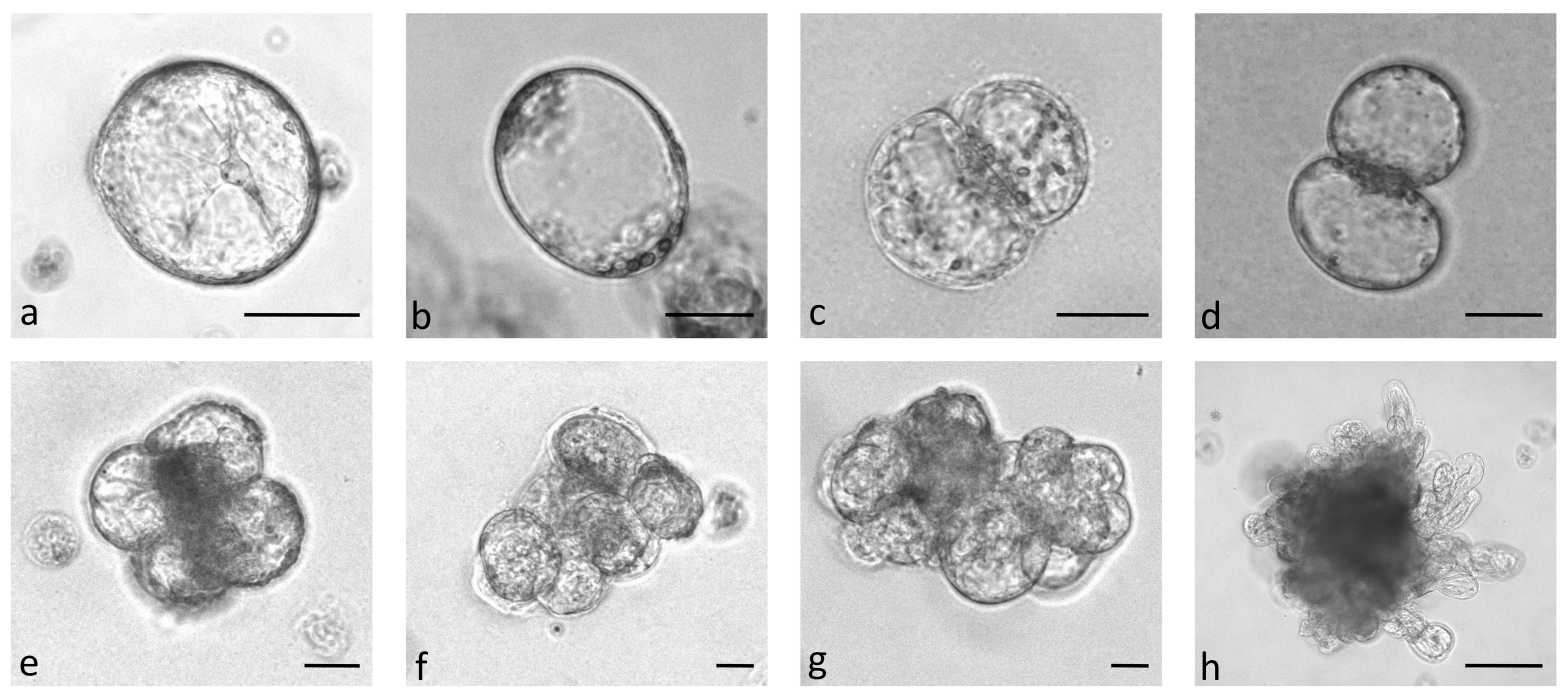
Stages of *Brassica oleracea* protoplast cultures: protoplasts with cytoplasmic strands **(A)**, oval protoplasts indicating cell wall re-synthesis **(B)**, occurrence of cell division plate **(C)**, first division of a cell **(D)**, multicellular structure with 4 **(E)**, 8 **(F)**, and more than 8 **(G)** cells, multicellular microcallus colony **(H)**. Scale bars represent either 25 µm **(A-G)** or 200 µm **(H)**.

In the first experiment, protoplasts of the four *B. oleracea* cultivars were immobilized in alginate discs at three different densities (2.5 x 10^4^ pp mL^-1^, 5 x 10^4^ pp mL^-1^, and 10 x 10^4^ pp mL^-1^). The selected densities were lower than the optimal ones that are generally used for culturing immobilized protoplasts (10^5^–10^6^ pp mL^-1^) (Davey et al., 2010; Reed and Bargmann, 2021) to determine the critical protoplast density that still allows minimal microcallus formation. Immediately after immobilization, protoplasts were of spherical shape with chloroplasts evenly distributed throughout the cell. By absorbing water, the protoplasts began to enlarge after 12 hours of culture, and 2 days after immobilization, transvacuolar cytoplasmic strands appeared with the nucleus in the center of the cell (**Figure 2A**). Two days later, their shape became irregular or oval, indicating cell wall resynthesis (**Figure 2B**), and the number of chloroplasts decreased. Also on the 4^th^ day, the first division plates became visible (**Figure 2C**), and the cells that had already started dividing (**Figure 2D**) were counted on the 5^th^ day of culture. Until the 15^th^ day of culture, different types of multicellular structures were visible in all treatments, which were categorized into the following groups: 2–4-cell structures (**Figure 2D, E**), 4–8-cell structures (**Figure 2F**), and structures with more than 8 cells (**Figure 2G**). Until the 20^th^ day of culture, the first star-like microcallus colonies were visible to the naked eye (**Figure 2H**), which were counted on the 30^th^ day of culture.

**Figure 3:**
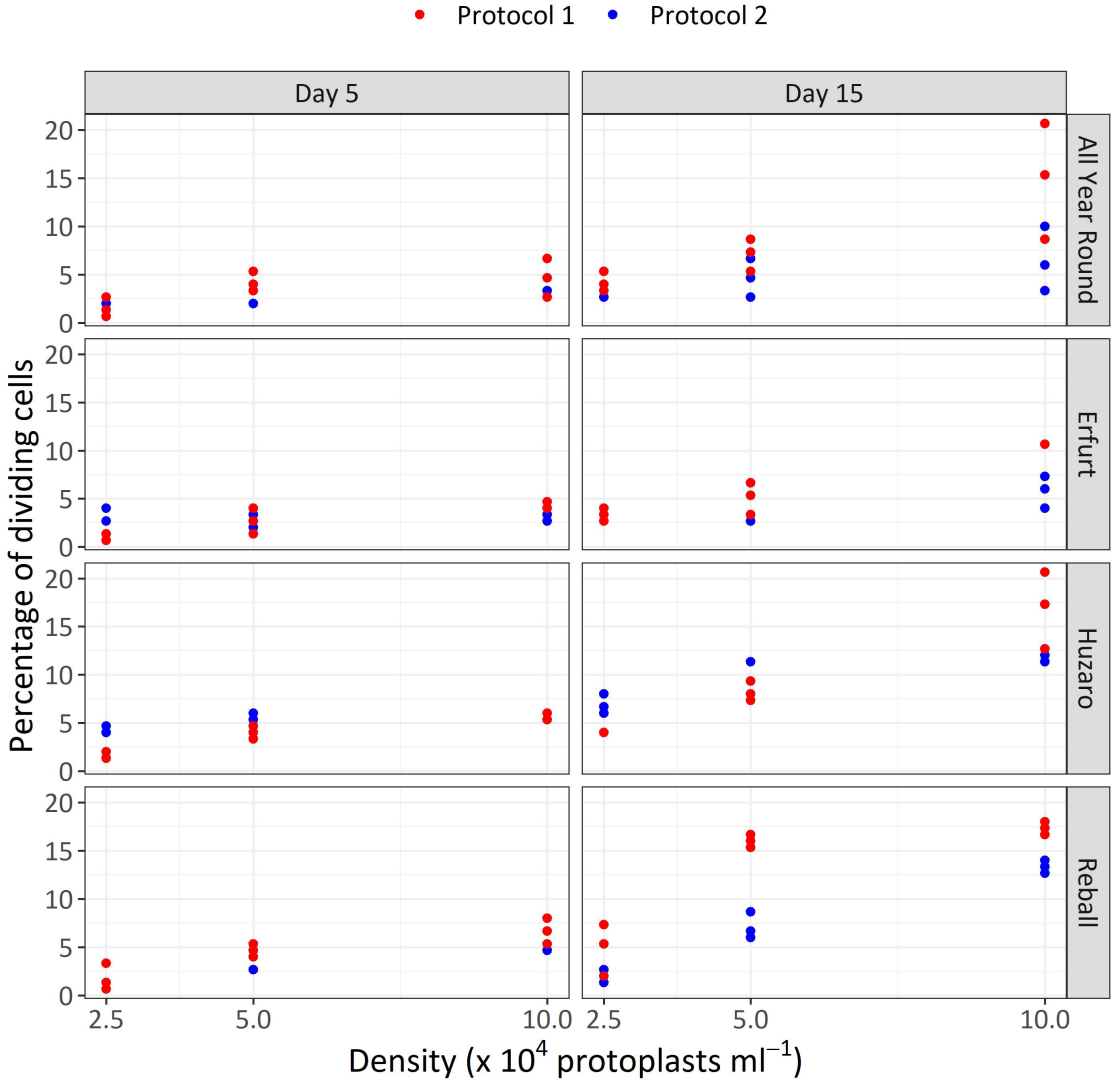
Percentages of dividing cells after 5 and 15 days of culture at three protoplasts densities (2.5, 5.0, and 10.0 x 10^4^ pp mL^-1^) and under two culture protocols (Protocol 1 and Protocol 2) for four *Brassica oleracea* cultivars (All Year Round, Erfurt, Huzaro, Reball). Each dot represents the result of a biological replicate.

The results of Experiment 1 (**Figure 3**) show that the percentages of dividing cells until the 5^th^ day of culture varied only slightly among the cultivars and protoplast densities and between the culture protocols (**Figure 3**, left side). On average, 0.9% to 6.6% of the immobilized protoplasts started dividing, and a positive trend with protoplast density was observed. The results obtained with Protocol 1 and Protocol 2 were comparable, and no clear differences could be observed among the cultivars. Almost all dividing structures contained 2 to 4 cells, demonstrating that in the first 5 days of culture, the cells divided once or twice. In four treatments, the discs contained also some 4–8-cell structures, and none of the dividing structures contained more than 8 cells.

Substantially (two- to three-fold) higher percentages of dividing cells were recorded at the 15^th^ day of culture (**Figure 3**, right side), when the differences between culture protocols and among protoplast densities became more pronounced. Protocol 1 resulted in higher division percentages for most cultivars at all protoplast densities, except for protoplasts of cultivar Huzaro at 2.5 and 5 x 10^4^ pp mL^-1^, where higher division percentages were recorded with Protocol 2 (**Table 1**). Division percentage was positively related with protoplasts density, whereas different cultivars exhibited similar results in all treatments.

**Table 1:** Mean values and corresponding 95% confidence intervals for percentages of dividing cells after 15 days of culture in Experiment 1.

Cultivar		All Year Round		Erfurt		Huzaro		Reball	
Density (pp mL^-1^)	Protocol	Mean (%)	95% CI (%)	Mean (%)	95% CI (%)	Mean (%)	95% CI (%)	Mean (%)	95% CI (%)
2.5 x 10^4^	1	4.2	2.7–6.6	3.3	2.0–5.5	4.0	2.5–6.3	4.9	3.2–7.4
	2	3.1	1.8–5.3	3.1	1.8–5.3	6.9	4.8–9.8	2.2	1.2–4.1
5 x 10^4^	1	7.1	5.0–10.1	5.1	3.4–7.7	8.2	6.0–11.3	16.0	12.7–20.2
	2	4.7	3.0–7.2	3.1	1.8–5.3	9.3	6.9–12.6	7.1	5.0–10.1
10 x 10^4^	1	14.9	11.7–18.9	10.7	8.0–14.2	16.9	13.5–21.1	17.3	13.9–21.6
	2	6.4	4.5–9.3	5.8	3.9–8.5	12.0	9.2–15.7	13.3	10.4–17.2

**Figure 4:**
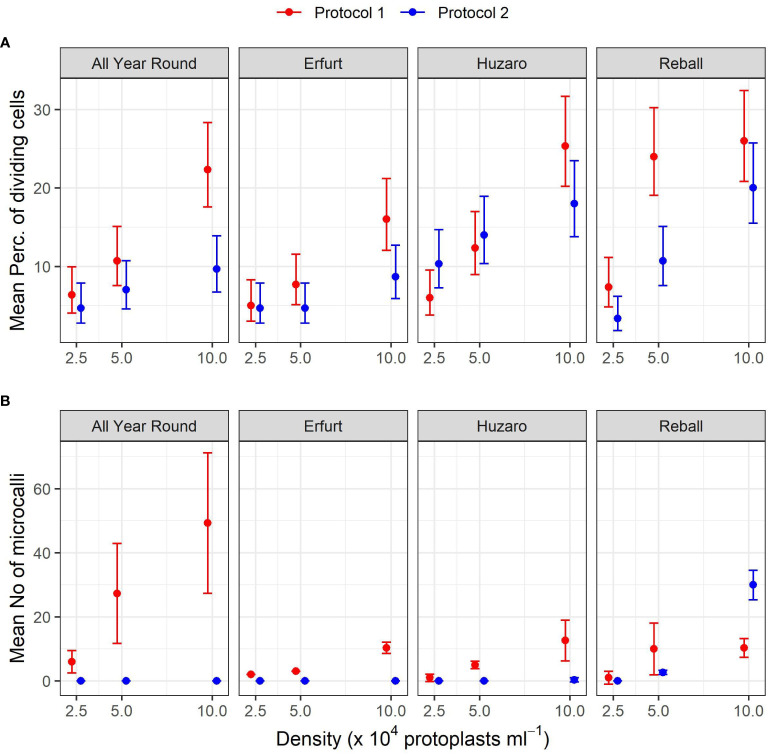
Mean percentages of dividing cells **(A)** and mean numbers of microcalli **(B)** obtained from protoplasts of four cultivars immobilized at three protoplast densities. The results are presented as mean values with corresponding 95% confidence intervals recorded after 15 **(A)** and 30 **(B)** days of culture under two different culture protocols, estimated with the Poisson regression model **(A)** and the negative binomial model **(B)**.

The mean percentages of dividing cells up to the 15^th^ day of culture were statistically analysed with the Poisson regression model, considering cultivar, protocol, density, and their interactions as fixed effects. The interaction between cultivar and protocol was statistically significant (p < 0.01), indicating that the influence of the protocol differed depending on the cultivar. All main factors were also statistically significant (p < 0.001). Due to the significance of the interaction, contrasts were set to compare the mean percentages of dividing cells among the different levels of three factors (**Figure 4A**).

As anticipated, Protocol 1 resulted in a higher mean percentage of dividing cells, but statistically significant higher percentages were found only at the highest protoplast density of 10 x 10^4^ pp mL^-1^ for both cauliflower cultivars, with fold changes of 2.3 (95% CI 1.5–3.6) for All Year Round and 1.9 (95% CI 1.2–3.0) for Erfurt. Among the cabbage cultivars, only Reball showed a significant improvement in the percentage of cell division with Protocol 1 at the two lower densities (**Supplementary Table 1**).

A comparison of the different protoplast densities confirmed significant differences. Higher ratios were found for protoplasts immobilized at the highest and lowest densities when cultured under Protocol 1. Statistically significant ratios between means of dividing percentages at densities 10 x 10^4^ pp mL^-1^ and 2.5 x 10^4^ pp mL^-1^ were found for all four cultivars (3.5, 3.2, 4.2, and 3.6 for All Year Round, Erfurt, Huzaro, and Reball, respectively). For Protocol 2, only cabbage cultivars Huzaro and Reball responded statistically significant to the increased density of immobilized protoplasts (**Supplementary Table 1**). Cells of Reball showed a 6-fold higher dividing percentage (95% CI 2.7–13.4) at the highest density when compared to the lowest one, which was also the highest calculated ratio between all significant contrasts. When comparing the highest and the medium densities, three cultivars showed significantly higher division percentages with Protocol 1, namely All Year Round, Erfurt, and Huzaro. Reball on contrary, showed a significant difference with Protocol 2. For all four cultivars, culturing protoplasts at 10 x 10^4^ pp mL^-1^ compared to 5 x 10^4^ pp mL^-1^ caused a 1.9-fold to 2.1-fold increase of the percentage of dividing cells at day 15. When comparing the medium and the lowest concentrations of immobilized protoplasts, significant contrasts were detected with Huzaro with Protocol 1 and Reball, with both protocols resulting in a 2.1-fold to 3.3-fold increase in the percentage of dividing cells (**Supplementary Table 1**).

Comparison of the cultivars showed that in general, cabbage had higher percentages of dividing cells than cauliflower, as indicated by the ratios below 1 when comparing the cauliflower cultivars All Year Round and Erfurt to the cabbage cultivars Huzaro and Reball. Differences between cauliflower and cabbage were significant at the medium and highest densities of immobilized protoplasts. Significant fold changes were also calculated between both cabbage cultivars, with different outcomes under different protocols and protoplast densities (**Supplementary Table 1**).

**Figure 5:**
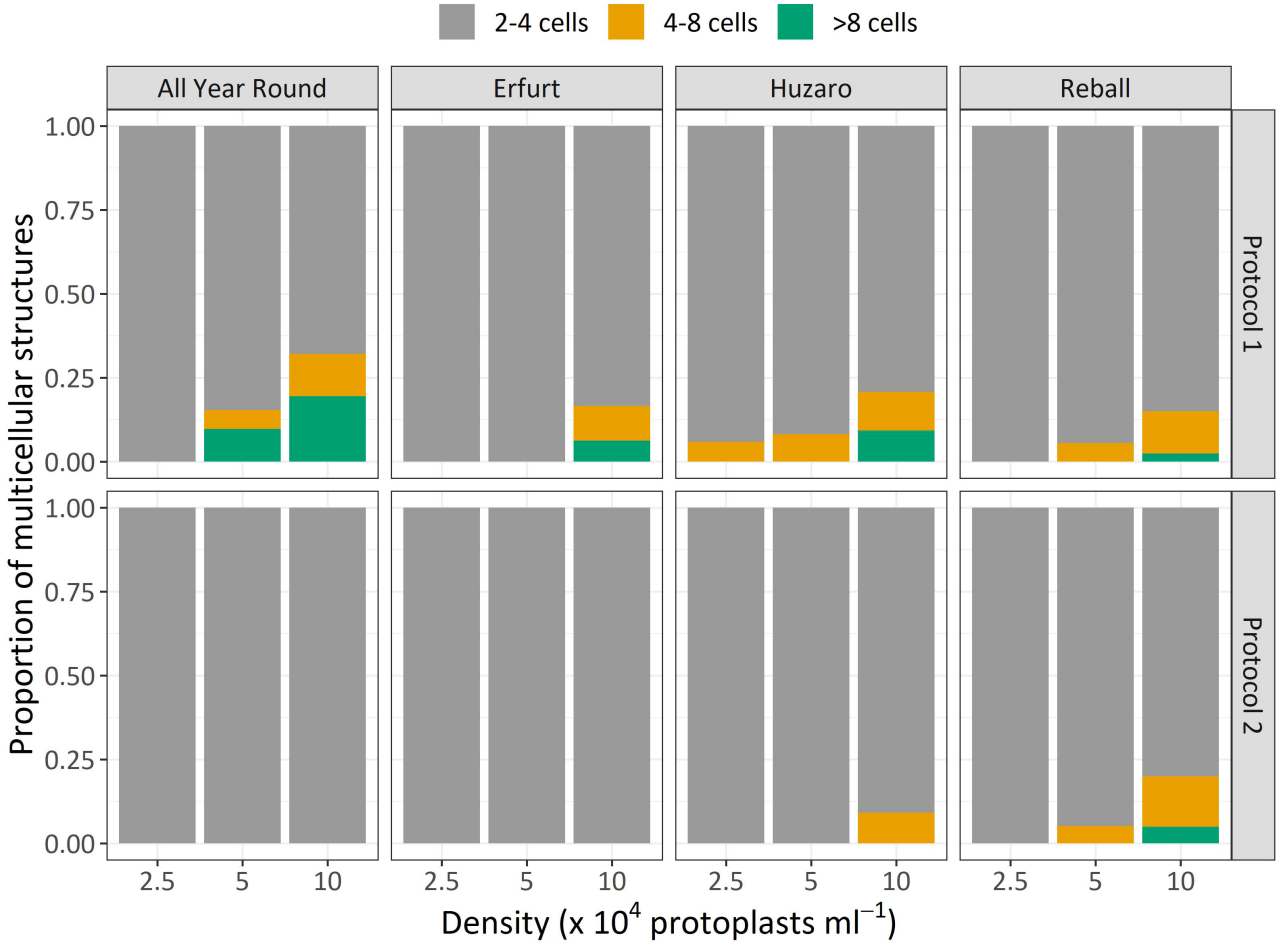
Mean proportions of multicellular structures according to the number of cells observed after 15 days of culture of four cultivars under two protocols and three protoplast densities.

The dividing structures observed at day 15 of culture were also categorized based on the number of cells they contained as an estimation of the number of cell divisions that had occurred. Compared to the results observed on day 5, at day 15, the discs contained a higher percentage of 4–8-cell structures and multicellular structures containing more than 8 cells. As shown in **Figure 5**, higher proportions of structures with 4–8 cells and more than 8 cells were obtained with Protocol 1 compared to Protocol 2 for almost all cultivars and protoplast densities. The only exception was noted with cultivar Reball, which, at the highest protoplast density (10 x 10^4^ pp mL^-1^), showed a slightly higher percentage of 4–8-cell (14.6%) structures and structures with more than 8 cells (5%) with Protocol 2 compared to Protocol 1 (11.5% and 3% for 4–8 cells and more than 8 cells, respectively). However, since also for Reball at the highest density, the percentage of multicellular structures with Protocol 1 (17.4%) was slightly higher than the percentage obtained with Protocol 2 (13.4%), the absolute number of structures with more than 4 cells was almost the same for Protocol 1 (3.8 per 150 protoplasts) and Protocol 2 (3.9 per 150 protoplasts). Cauliflower cultivars All Year Round and Erfurt did not form any structures with more than 4 cells under Protocol 2, irrespective of the protoplast density.

### 3.3 Exp 1: Microcallus formation

The effects of the factors cultivar, density, and protocol were quantified 30 days after the start of the culture by counting the number of microcallus colonies larger than 0.5 mm that had formed in each alginate disc. The 0.5-mm threshold value was chosen based on our preliminary experiments, which demonstrated that microcalli smaller than 0.5 mm do not survive subcultivation to solid shoot regeneration medium (data not shown). All alginate discs within this study were prepared from 100 µL of a protoplast-alginate mixture, but they contained different numbers of protoplasts depending on the tested density. This way, discs at the protoplast density of 2.5 x 10^4^ pp mL^-1^ contained 2,500 protoplasts, those with a density of 5 x 10^4^ pp mL^-1^ contained 5,000 protoplasts, and those with a density of 10 x 10^4^ pp mL^-1^ contained 10,000 protoplasts. Therefore, the number of regenerated microcalli per disc did not only influence the concentration of immobilized protoplasts but also the absolute number of protoplasts, i.e., the number of potential sources of divisions. The numbers of obtained microcalli were therefore normalized to 10,000 cultured protoplasts.

The results are presented in **Table 2** and **Figure 4B**, with mean values and corresponding 95% confidence intervals, indicating that a minimum of 0.0 and a maximum of 49.3 microcallli formed per 10,000 cultured protoplasts.

**Table 2:** Mean values and corresponding 95% confidence intervals for number of microcalli per 10,000 cultured protoplasts after 30 days of culture in Experiment 1.

Cultivar		All Year Round		Erfurt		Huzaro		Reball	
Density (pp mL^-1^)	Protocol	Mean	95% CI	Mean	95% CI	Mean	95% CI	Mean	95% CI
2.5 x 10^4^	1	6.0	3.6–10.1	2.0	0.9–4.6	1.0	0.3–3.2	1.0	0.3–3.2
	2	0.0	0.0–∞	0.0	0.0–∞	0.0	0.0–∞	0.0	0.0–∞
5 x 10^4^	1	27.3	19.8–37.7	3.0	1.5–6.0	5.0	2.9–8.7	10.0	6.5–15.4
	2	0.22	0.0–∞	0.0	0.0–∞	0.0	0.0–∞	2.7	1.3–5.5
10 x 10^4^	1	49.3	37.0–65.7	10.3	6.8–15.8	12.7	8.5–18.8	10.3	6.8–15.8
	2	0.00	0.0–∞	0.0	0.0–∞	0.3	0.0–2.4	30.0	21.9–41.1

The numbers of developed microcalli were statistically analysed with the negative binomial regression model considering cultivar, protocol, density, and their interactions as fixed effects. The interactions between cultivar and protocol, cultivar and density, and density and protocol were statistically significant (p < 0.001), and all main factors showed statistically significant effects (p < 0.001). Specific contrasts were set to compare the mean numbers of microcalli (**Figure 4B**) among different levels of the three factors. The number of microcalli was highest at the highest protoplast density of 10 x 10^4^ pp mL^-1^, with a 5.2-fold to 10.3-fold higher production of microcalli compared to that of protoplasts cultured at the lowest density. The fold changes differed depending on the cultivar but were significant for all cultivars cultured under Protocol 1. Comparison of the highest and the medium density revealed that cultivars All Year Round, Erfurt, and Huzaro produced significantly more microcalli under Protocol 1, whereas Raball also produced more microcalli at the highest density than at the medium one, albeit under Protocol 2 (**Supplementary Table 2**).

At the highest protoplast density, Protocol 1 outperformed Protocol 2 with cells of Huzaro, with a fold change of 38 (95% CI 5–275). Cells of cultivar Reball produced more microcalli with Protocol 1 at the density of 5 x 10^-4^ pp mL^-1^ (3.8 fold change, 95% CI 1.6–8.8) and less microcalli with Protocol 1 at the highest density of 10 x 10^-4^ pp mL^-1^ (34% that at Protocol 3, 0% CI 20%–58%) (**Supplementary Table 2**).

In contrast to the results observed at day 15, when cabbage cultivars showed a higher percentage of dividing cells than cauliflower cultivars (**Supplementary Table 1**), at day 30, the cauliflower cultivar All Year Round produced significantly more microcalli than both cabbage cultivars and showed the highest dividing potential when cultured under Protocol 1. It produced from 3.9-fold (95% CI 2.1–7.4) to 6.0-fold (95% CI 1.1–31.6) more microcalli than Huzaro and from 2.73-fold (95% CI 1.4–5.5) to 6.0-fold (95% CI 1.1–31.6) more microcalli than Reball at different protoplast densities. It even produced more microcalli than cultivar Erfurt at the highest (4.8-fold, 95% CI 2.4–9.4) and medium (9.1-fold, 95% CI 3.3–24.9) protoplast densities (**Supplementary Table 2**). These results can be explained by the highest proportion of multicellular structures with more than 4 cells, which were observed for cultivar All Year Round under Protocol 1 (**Figure 5**).

### 3.4 Exp 1: Shoot regeneration

**Figure 6:**
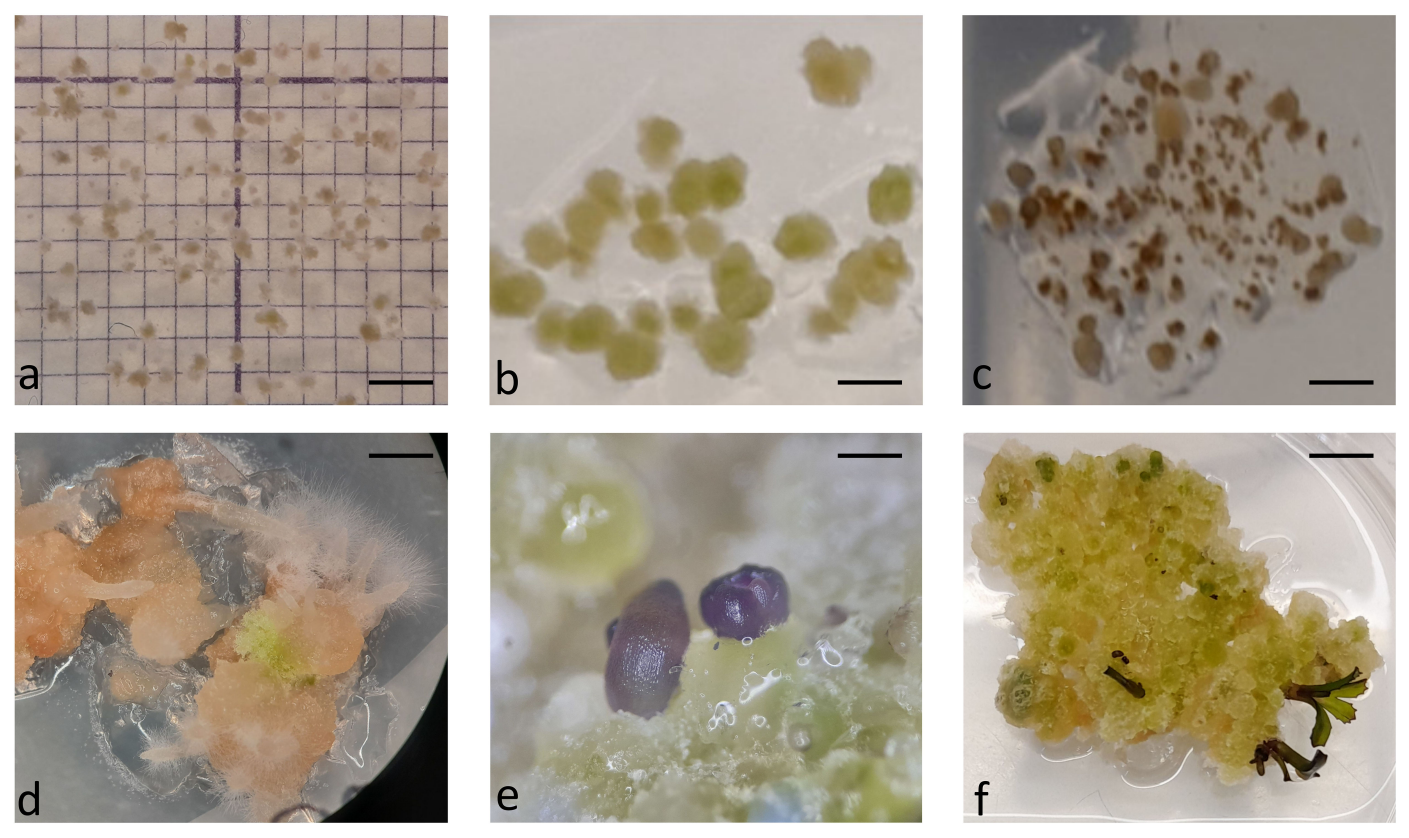
Callus development and shoot regeneration after transfer to solid medium: microcallus colonies in alginate disc at the day of transfer to solid shoot regeneration medium **(A)**, further divisions of cells and callus growth after 2 weeks of culture on regeneration medium **(B)**, browning of microcalli smaller than 0.5 mm **(C)**, white proliferating callus that did not develop shoots but only roots **(D)**, differentiation of Reball callus cells into shoot primordia after 4 weeks on regeneration medium **(E)**, and further development of shoots after 6 weeks on regeneration medium **(F)**. Scale bars represent either 2 mm **(A-C)** µm, 5 mm **(D-F)**.

After determining the number of microcalli formed in discs cultured in liquid medium for 30 days, whole alginate discs containing microcalli (**Figure 6A**) were transferred to solid regeneration media according to the Protocols. For Protocol 1, the discs were transferred directly to solid medium E, whereas for Protocol 2, the discs were placed on filter papers moistened with liquid CPPD medium placed on solid Murashige and Skoog medium supplemented with 1 mg L^-1^ BAP. The microcalli began to grow at an accelerated rate at two weeks after transfer to solid media, irrespective of the protocol (**Figure 6B**). However, browning of microcalli was observed in numerous discs, impeding shoot regeneration. Shortly after transfer to solid medium E, microcalli smaller than 0.5 turned brown in discs with lower initial protoplast densities (2.5 and 5 x 10^4^ pp mL^-1^) (**Figure 6C**), and after 4 weeks, when fast callus growth ceased, also some well-developed calli from discs with the highest initial protoplast density (10 x 10^4^ pp mL^-1^) turned brown. Until the ninth week after transfer to E-medium (based on Protocol 1), all calli of the Huzaro cultivar were brown, along with 22% of discs of the Erfurt cultivar and 33% of discs of the All Year Round cultivar; no browning was observed for the Reball cultivar. The microcalli from the remaining discs either remained green without shoots, or a white-brownish callus developed that produced only roots and hairy roots (**Figure 6D**). Callus browning also occurred when protoplast discs were transferred to solid medium according to Protocol 2. In these cases, the calli from half of the discs turned brown, and those from the other half of the discs either remained green and friable without shoots, or the calli from Reball became compact with globular structures (**Figure 6E**) and later developed shoots (**Figure 6F**). The first shoot primordia (**Figure 6E**) appeared 4 weeks after transferring the discs to Murashige and Skoog medium with BAP, and the first shoots were ready for outgrowth and rooting after 6 weeks (**Figure 6F**). Shoots developed only from calli of the cultivar Reball as this was the only cultivar that had developed sufficient microcalli until the 30^th^ day of culture under Protocol 2. The red phenotype of cabbage Reball (*B. oleracea* var. *capitata* forma *rubra*) was visible already at the phase of shoot primordia (**Figure 6E**). Shoots developed only from the discs with the highest protoplast density, from which, on average, 2.4 shoots per disc developed.

### 3.5 Exp 2: Mitogenic activity of PSK

Since Experiment 1 demonstrated that the percentage of dividing cells and the ability to form microcalli largely depends on the initial protoplast density and that even the extremely low protoplast density of 2.5 x 10^4^ pp mL^-1^ enabled at least minimum percentages of dividing cells and microcallus formation, this density was primarily used in the following experiment, together with the highest density for comparison, because it resulted in significantly higher cell division rates. Experiment 2 aimed at elucidating the effect of the addition of 0.1 µM PSK into the liquid medium in which protoplast-alginate discs were cultured for the first 10 days. The PSK is a known mitogenic factor that might compensate low concentrations of various mitogenic factors in cultures with low protoplast density, consequently enhancing cell division. To evaluate whether PSK supplementation is genotype-dependent, all four cultivars were included in Experiment 2. Based on the results of Experiment 1, where Protocol 1 produced more dividing cells and microcalli, whereas Protocol 2 better stimulated the further division of callus cells and their differentiation into shoots, both protocols were used in Experiment 2 (**Figure 1**). Culture of protoplasts and cells with or without PSK supplementation followed Protocol 1 until the 30^th^ day of culture, when the formed microcalli were subcultured to solid regeneration medium as in Protocol 2.

**Figure 7:**
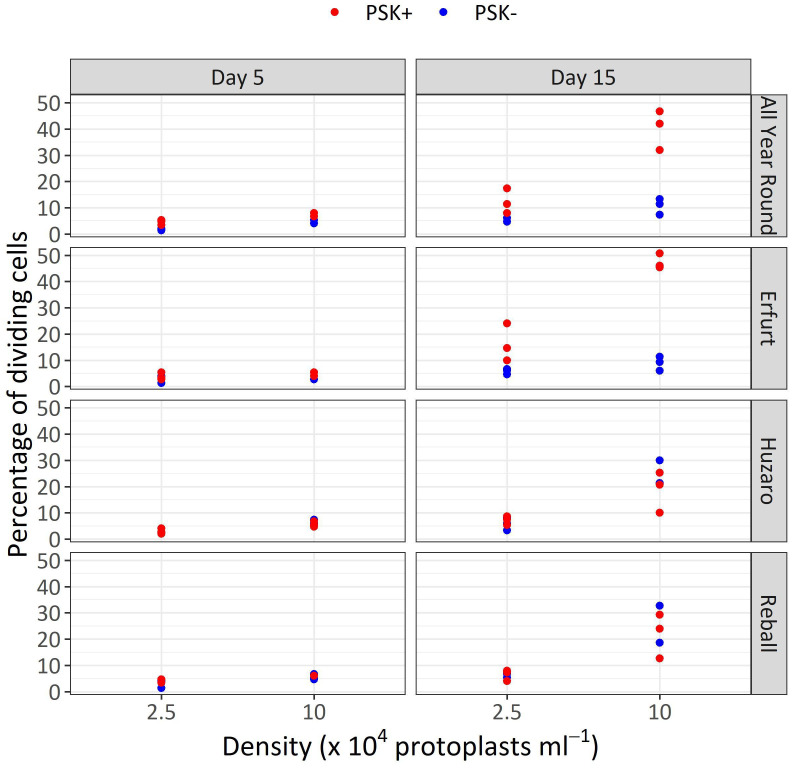
Percentages of dividing cells after 5 and 15 days of culture at two protoplasts densities. All cultures followed Protocol 1, and half of the media were supplemented with 0.1 µM PSK. Each dot represents the result of a biological replicate.

After 5 days of culture, a stimulatory effect of PSK on percentages of dividing cells was indicated, although the percentages of dividing cells of all cultivars remained low (**Figure 7**). Almost all multicellular structures contained 2–4 cells, and only in three treatments, some 4–8-cell structures were detected; none of the structures had more than 8 cells, similar to the findings at day 5 in Experiment 1. Considerably higher percentages of dividing cells were again observed after 15 days of culture, when the mitogenic activity of PSK increased the percentages of dividing cells in both cauliflower cultivars All Year Round and Erfurt. The PSK stimulated the division of cauliflower cells cultured at both initial protoplast densities, but the effect was more pronounced at the higher density (**Figure 7**; **Table 3**).

**Table 3:** Mean values and corresponding 95% confidence intervals for the percentages of dividing cells after 15 days of culture in Experiment 2.

Cultivar		All Year Round		Erfurt		Huzaro		Reball	
Density (pp ml^-1^)	Supplementation	Mean	95% CI	Mean	95% CI	Mean	95% CI	Mean	95% CI
2.5 x 10^4^	PSK-	5.6	3.0–10.4	5.8	3.1–10.7	5.8	3.1–10.7	6.7	3.8–11.8
	PSK+	12.2	8.0–18.7	16.2	11.2–23.4	7.1	4.1–12.4	6.4	3.6–11.5
10 x 10^4^	PSK–	10.7	6.8–16.8	8.9	5.4–14.6	20.4	14.7–28.3	21.3	15.5–29.4
	PSK+	40.2	31.9–50.8	47.3	38.2–58.7	18.7	13.3–26.3	22.0	16.1–30.1

Analysis of variance for the quasi Poisson regression model showed a statistically significant interaction between the factors cultivar and PSK supplementation (p < 0.001) and a significant influence of the main factors cultivar (p < 0.01), PSK supplementation (p < 0.001), and protoplast density (p < 0.001). For both cauliflower cultivars at both protoplast densities, supplementation of 0.1 µM PSK in liquid medium for 10 days enhanced the percentage of cell division in All Year Round by 2.2-fold (95% CI 1.0–4.7) and 3.8-fold (95% CI 2.3–6.3) at densities of 2.5 and 10 x 10^4^ pp mL^-1^, respectively. Cells of the cultivar Erfurt responded even more pronounced to the addition of PSK and increased their division frequencies by 2.8-fold (95% CI 1.4–5.8) and 5.3-fold (95% CI 3.1–9.1) at the lower and higher density, respectively (**Supplementary Table 3**).

Contrast analysis for the comparison of culture densities showed that the cauliflower cultivars produced significantly higher percentages of dividing cells with the addition of PSK, with a 3.3-fold (95% CI 2.0–5.3) increase for All Year Round and a 2.9-fold (95% CI 1.9–4.5) increase for Erfurt. In contrast, the cabbage cultivars showed significant improvements in percentages of dividing cells in media with or without PSK. At the high density, 2.6-fold (95% CI 1.4–5.0) to 3.5-fold (95% CI 1.8–7.1) more cells divided than at the low density of immobilized protoplasts. With the addition of PSK, the cauliflower cultivars outperformed the cabbage cultivars at the highest density, with fold changes from 1.8-fold (95% CI 1.1–3.1) to 2.5-fold (95% CI 1.5–4.3) (**Supplementary Table 3**).

**Figure 8:**
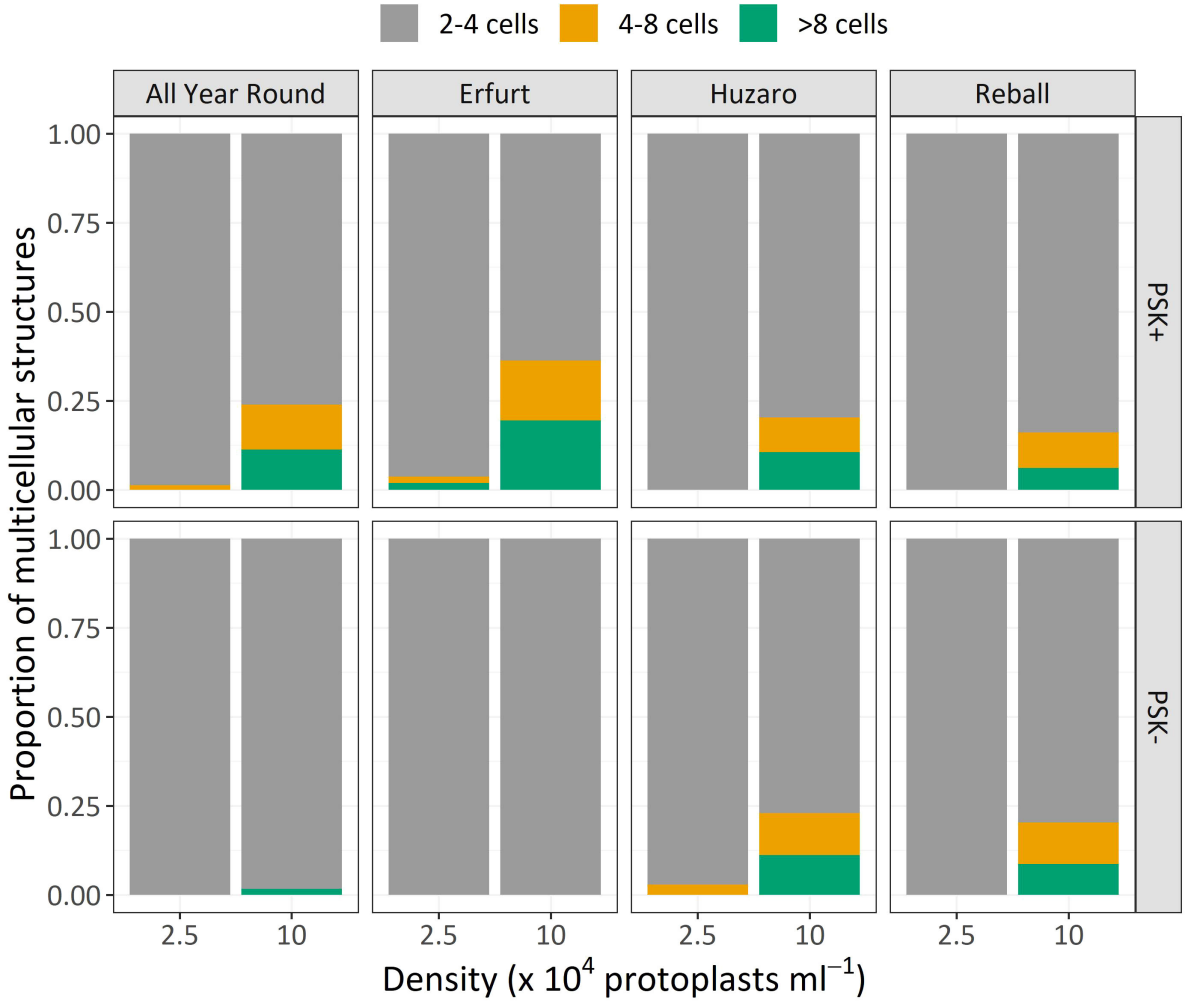
Mean proportions of multicellular structures according to the number of cells observed after 15 days of culture of four cultivars cultured at two protoplast densities with or without supplementation of 0.1 µM PSK.

Similar to Experiment 1, the multicellular structures on day 15 were categorized based on the number of cells present in each structure (**Figure 8**). The proportions of multicellular structures showed that in cauliflower cultivars, PSK stimulated cell division, resulting in a higher proportion of structures with more than 4 cells in the first 15 days of culture. For cabbage cultivars, no such differences were observed.

### 3.6 Exp 2: Effect of PSK on microcallus formation

**Figure 9:**
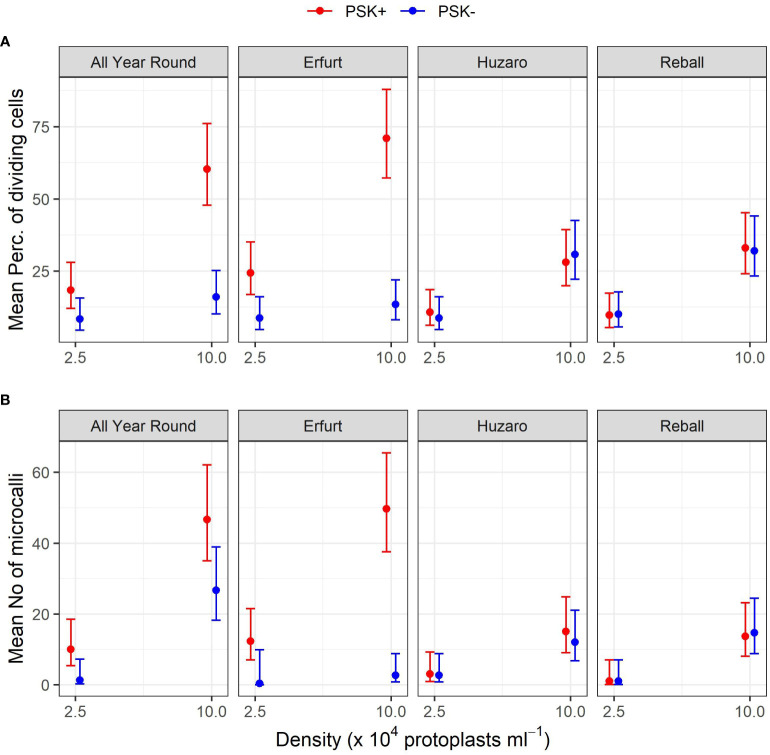
Mean percentages of dividing cells **(A)** and mean numbers of microcalli **(B)** obtained from protoplasts of four cultivars immobilized at two protoplast densities. The results are presented as mean values with corresponding 95% confidence intervals recorded after 15 a and 30 **(B)** days of culture under Protocol 1 with or without 0.1 µM PSK in liquid medium, estimated with the quasi Poisson regression model **(A)** and the negative binomial model **(B)**.

The mean microcallus numbers per 10,000 protoplasts in Experiment 2 ranged from 0.3 to 49.7 (**Table 4**; **Figure 9B**), similar to the results obtained in Experiment 1 (**Table 2**; **Figure 4B**).

**Table 4:** Mean values and corresponding 95% confidence intervals for the number of microcalli per 10,000 cultured protoplasts after 30 days of culture in Experiment 2.

Cultivar		All Year Round		Erfurt		Huzaro		Reball	
Density (pp ml^-1^)	Supplementation	Mean	95% CI	Mean	95% CI	Mean	95% CI	Mean	95% CI
2.5 x 10^4^	PSK-	1.3	0.2–7.3	0.3	0.0–9.9	2.7	0.8–8.8	1.0	0.1–7.1
	PSK+	10.0	5.4–18.6	12.3	7.1–21.5	3.0	1.0–9.3	1.0	0.1–7.1
10 x 10^4^	PSK-	26.7	18.3–39.0	2.7	0.8–8.8	12.0	6.8–21.1	14.7	8.8–24.4
	PSK+	46.7	35.0–62.1	49.7	37.6–65.6	15.0	9.1–24.9	13.7	8.0–23.2

The mean number of microcalli at the low protoplast density and without PSK were low and similar among the cultivars and to those obtained with cabbage cultivars with the addition of PSK. Substantially higher mean values were obtained in cauliflower cultivars with the addition of PSK, especially at the high protoplast density.

Analysis of variance based on the negative binomial regression model revealed that cultivar, PSK supplementation, protoplast density, and interaction between cultivar and supplementation were highly statistically significant (p < 0.001). A further analysis of contrasts showed that the addition of PSK had a stronger stimulatory effect on discs with low initial protoplast density, which was in contrast to the results observed for day 15. Discs of cultivar All Year Round produced 1.8-fold (95% CI 1.1–2.8) more microcalli at the high density and 7.5-fold (95% CI 1.2–45.6) more microcalli at the low density when PSK was added to the culture medium. Similarly, discs of Erfurt produced 18.6-fold (95% CI 5.4–63.7) and 37-fold (95% CI 1–1147) more microcalli at the high and low densities, respectively. The wide CI range for Erfurt protoplasts at low density indicates a low estimate precision. These results were unexpected given that the PSK was present only for the first 10 days; apparently, the PSK had a strong effect also after further 20 days of culture without PSK (**Supplementary Table 4**).

Significantly higher numbers of microcalli were obtained at the high initial protoplast density compared to the low one for cultivars All Year Round, Huzaro, and Reball under both conditions (with or without PSK), whereas for cultivar Erfurt, a significant difference was confirmed only if PSK was added to the medium. With the addition of PSK, the cauliflower cultivars outperformed the cabbage cultivars in the number of microcalli at the highest density, with fold changes from 3.11-fold (95% CI 1. 5–6.7) to 3.6-fold (95% CI 1.7–8.0) (**Supplementary Table 4**).

### 3.7 Exp 2: Effect of PSK on callus differentiation and shoot regeneration

Since there was no shoot regeneration on solid medium according to Protocol 1 in Experiment 1, in Experiment 2, further culture on regeneration medium followed Protocol 2. Several combinations of the presence or absence of PSK in the liquid and solid medium were tested. In the absence of PSK in liquid medium, few shoots developed later on the solid one, except for a few discs at the highest protoplast density of the cultivar Reball. With the presence of 0.1 µM PSK in solid medium, a mean of 1.5 ± 0.8 shoots per disc developed. Numerous shoots were obtained after 4–6 weeks on solid medium from discs with the higher protoplast density if they had initially been cultured in liquid medium with PSK for 10 days. In these cases, cultivar Erfurt showed the highest number of regenerated shoots among the four cultivars. At the highest protoplast density, its callus did not form any shoots without PSK, at least in the liquid medium; on average, 3.1 ± 1.2 shoots per disc were observed when PSK was present only in the liquid medium and 13.8 ± 2.7 shoots per disc when PSK was present in both the liquid and the solid medium. A similar trend was noted at the low protoplast density, where no shoots were regenerated without PSK at least in liquid medium, 0.7 ± 0.3 shoots per disc with PSK only in liquid medium, and 3.1 ± 0.3 shoots per disk if PSK was present both in the first 10 days in liquid medium and later on in solid shoot regeneration medium.

### 3.8 Exp 3: Effect of PSK on microcallus formation from transformed protoplasts

Experiment 3 was designed to verify whether PSK supplementation can improve regeneration from protoplasts previously transformed with PEG (**Figure 1**). For this part of the study, only protoplasts of the cultivar Erfurt were used as they were most responsive to PSK in Experiment 2 (**Supplementary Table 4**) and their viability after PEG-mediated transformation dropped substantially in our previous experiments (data not shown). They were cultured at an initial density of 40 x 10^4^ pp mL^-1^, which is considered an optimal density (Sheng et al., 2011; Kiełkowska and Adamus, 2012, 2019, 2021), following Protocol 1 for 30 days when the number of microcalli was determined. As a control, untransformed protoplasts were also immobilized and cultured as the transformed ones.

**Figure 10:**
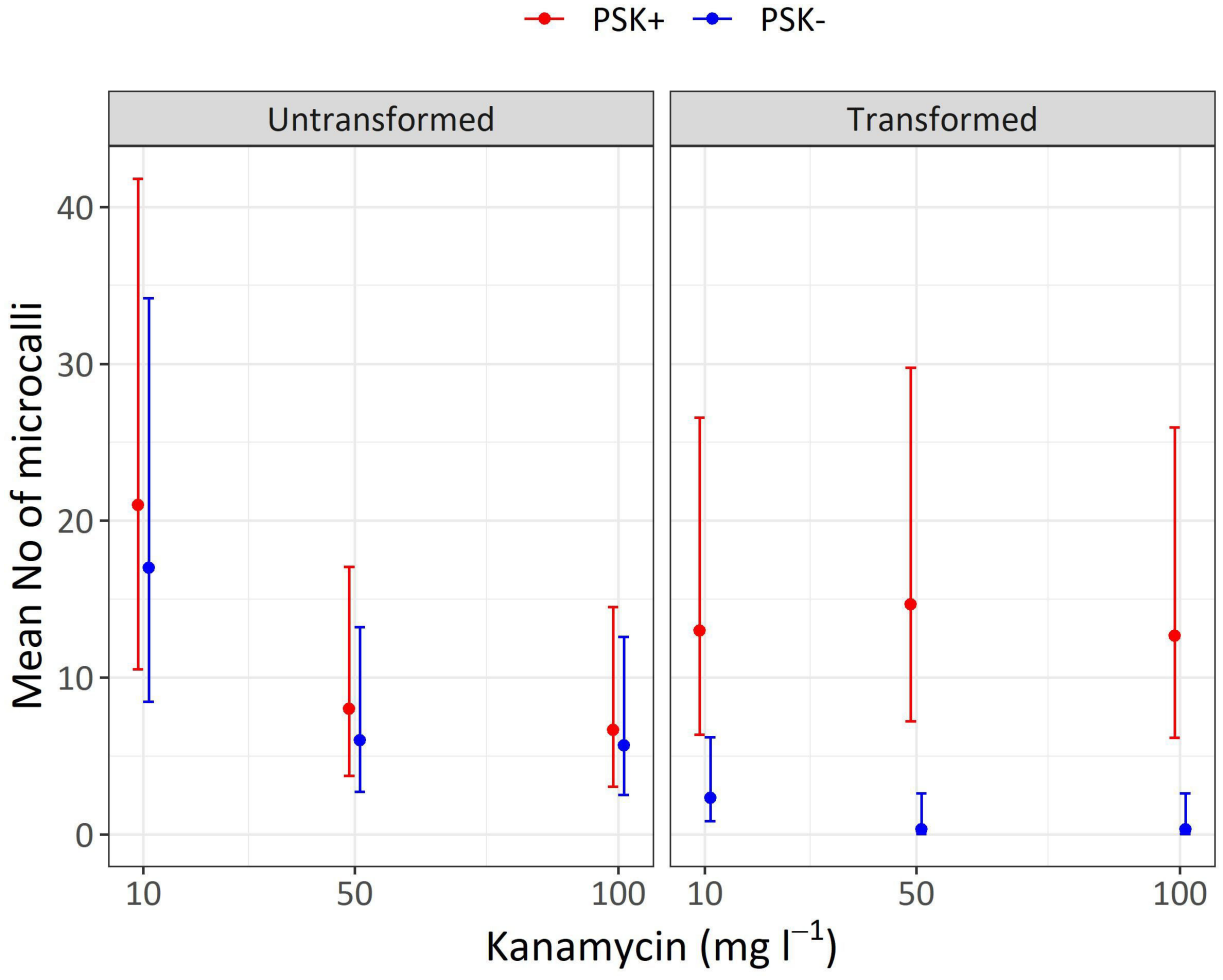
Mean numbers of microcalli obtained from untransformed and transformed protoplasts immobilized in alginate discs and cultured in liquid media supplemented with three concentrations of kanamycin. The results are presented as mean values with corresponding 95% confidence intervals recorded after 30 days of culture under Protocol 1 with or without 0.1 µM PSK in liquid medium.

Staining of alginate discs with FDA revealed that PEG-mediated transformation had a strong negative effect on protoplast viability. Whilst 81.9 ± 1.6% of untransformed protoplasts was still viable 48 hours after immobilization in alginate discs, the percentage of viable protoplasts decreased to 32.5 ± 1.4% when they had been treated with PEG. Later on, on the 30^th^ day of culture, the mean number of developed microcalli per disc ranged between 0.3 and 21.0 and varied based on the concentration of the selective antibiotic kanamycin, supplementation with PSK, and whether the protoplasts were transformed or not (**Table 5**; **Figure 10**).

**Table 5:** Mean values and corresponding 95% confidence intervals for number of microcalli per disc after 30 days of culture in Experiment 3.

Cultivar	Kanamycin (mg L^-1^)	10		50		100	
Transformation	Supplementation	Mean	95% CI	Mean	95% CI	Mean	95% CI
Untransformed	PSK-	17.0	8.5–34.2	6.0	2.7–13.2	5.7	2.5–12.6
	PSK+	21.0	10.6–41.8	8.0	3.8–17.1	6.7	3.1–14.5
Transformed	PSK-	2.3	0.9–6.2	0.3	0.0–2.6	0.3	0.0–2.6
	PSK+	13.0	6.4–26.6	14.7	7.2–29.7	12.7	6.2–25.9

Analysis of variance showed that the interaction of transformation and supplementation was statistically significant (p < 0.001), and the main factors also showed significant influences: supplementation (p < 0.001), transformation (p < 0.01), kanamycin concentration (p < 0.05). Treatment of protoplasts with PEG had a strong negative influence on the number of formed microcalli since untransformed protoplasts produced 7.3-fold (95% CI 2.2–24.3) more microcalli than transformed ones when cultured with 10 mg L^-1^ kanamycin, 18-fold (95% CI 2–1634) more microcalli when cultured with 50 mg L^-1^ kanamycin, and 17-fold (95% CI 2–155) more microcalli when cultured with 100 mg L^-1^ kanamycin (**Supplementary Table 5**). The results showed that PEG treatment had a stronger effect on the number of microcalli than culture under selective pressure of the antibiotic kanamycin. At the lower concentration of 10 mg L^-1^, even the untransformed protoplasts efficiently formed microcalli (17 and 21 microcalli per disc), and the number of microcalli decreased only at the higher antibiotic concentrations of 50 and 100 mg L^-1^ (from 5.7 to 8.0 microcalli per disc). The unexpectedly high number of microcalli from untransformed protoplasts on selective pressure could be explained by the binding of the positively charged kanamycin to the negatively charged alginate, which probably led to decrease in antibiotic activity. By increasing the concentration of the antibiotic, the numbers of microcalli were reduced, but even at the highest concentration, up to 6.7 microcalli formed from untransformed protoplasts (**Table 5**; **Figure 10**).

The drastic decrease in viability and, consequently, the number of microcalli from transformed protoplasts was efficiently compensated with the addition of PSK, which enabled a 5.6-fold (95% CI 1.7–18.8) increase in the number of microcalli at the lowest kanamycin concentration of 10 mg L^-1^. An even higher mitogenic effect was recorded when the transformed protoplasts were cultured at the two higher kanamycin concentrations, which on contrary, caused a substantial decrease in the number of microcalli from untransformed protoplasts. The number of microcalli from transformed protoplasts was 44-fold (95% CI 5–389) higher in cultures with 50 mg L^-1^ kanamycin and 38-fold (95% CI 4–337) higher in cultures with 100 mg L^-1^ kanamycin (**Supplementary Table 5**). The latter results indicate that the protoplasts were efficiently transformed, and the *nptII* gene was conferring resistance to kanamycin. Therefore, the highest numbers of microcalli at high kanamycin concentrations were sourced from transformed and selected cells.

The *GFP* fluorescence in cells, multicellular structures, and microcalli in discs could not be reliably monitored due to the non-translucence of alginate and the autofluorescence emitted from cultured cells. To unequivocally confirm that the regenerated microcalli from transformed protoplasts cultured under high selective pressure are indeed transformants, molecular characterization and *GFP* expression analysis should be performed on regenerated shoots, which is beyond the scope of this study.

## 4 Discussion

Since the discovery of the genome editing technology CRISPR/Cas9 a decade ago, protoplasts have regained their role as promising targets for genetic manipulation. The lack of a cell wall enables the fast and efficient introduction of heterologous macromolecules (DNA, RNA, or proteins) via PEG-mediated transformation or electroporation, and various outcomes can be designed. They are nowadays routinely used for testing efficiencies of gRNAs, new genome editing technologies such as base and prime editing, new Cas9 or other enzyme types, and for homology-directed repair. Moreover, as shown also in our study on *Brassica* protoplasts (Murovec et al., 2018), the introduction of CRISPR/Cas9 as ribonucleoprotein complexes into plant protoplasts can efficiently modify endogenous genes without the integration of foreign DNA into the host cell and genome.

However, as excellently summarized in the review by Reed and Bargmann (2021), the use of protoplasts for plant genome editing is still limited by the inability of several plant species to efficiently regenerate plants from treated protoplasts. Regeneration protocols vary among species and are, overall, relatively inefficient. Moreover, as they are highly dependent on the genotype of the plant, the regeneration protocols must be optimized even for individual genotypes within otherwise susceptible plant species.

The present study was therefore designed to determine whether supplementation of culture media with PSK could improve protoplast regeneration after PEG-mediated transformation. It was divided into three experiments focused on different experimental questions (**Figure 1**). The first experiment tested the influence of the genotype, initial protoplast density, and culture protocol on the regeneration of untransformed protoplasts. *In vitro* seedlings of four cultivars belonging to two morphotypes of *B. oleracea* were used in this experiment, namely cultivars All Year Round and Erfurt of the cauliflower morphotype and cultivars Huzaro and Reball of the cabbage morphotype. The experiment showed statistically significant differences among the genotypes, with cabbage cultivars having a higher percentage of dividing cells by day 15 of culture (**Figure 3**, **4A**, **Table 1**; **Supplementary Table 1**), but the trend reversed with further cultivation. At 30 days of culture, the cauliflower cultivar All Year Round produced statistically significantly higher numbers of microcalli than both cabbage cultivars and even the other cauliflower cultivar Erfurt (**Figure 4B**; **Table 2**). The mean numbers of All Year Round microcalli were up to 6-times higher than those of the microcalli of the cabbage cultivars Huzaro and Reball (**Supplementary Table 2**). The influence of the genotype was significant also in the second experiment, in which half of the treatments involved the supplementation of liquid medium with 0.1 µM PSK for the first 10 days of culture. In such case, when PSK was added, the cauliflower cultivars outperformed the cabbage cultivars already at day 15 in the percentage of dividing cells (**Figure 7**, **9A**; **Table 3**) and later at day 30 in the number of developed microcalli (**Figure 9B**; **Table 4**). Since at the day of protoplast isolation, all cultivars showed high and comparable protoplast viability, the observed differences in percentages of dividing cells and microcallus formation were determined to be caused by genotypic differences among cultivars and not by initial differences in protoplast viability.

Within Experiment 1, we also wanted to determine which of the two culture protocols used for different *Brassica species* in the past is more suitable for culturing protoplasts of cauliflower and cabbage cultivars. Protocol 1 originated from a protocol developed by Pelletier and colleagues (Pelletier et al., 1983) to culture cybrids of *B. napus* and *B. campestris* to mitigate the low chlorophyll content of *B. napus* lines with cytoplasmic male sterility. The protocol involved a sequence of six media used for the incubation of alginate discs, depending on the stage of protoplast development: the first medium (B) allows the induction of cell division, the second (C) accelerates cell division, the third (D) allows the development of microcalli, the fourth allows callus growth and shoot regeneration (E), and the fifth (F) facilitates elongation and shoot growth. The last medium (G) enables shoot rooting. Within Protocol 2, the composition of the culture medium CPP does not change during media refreshments every 10 days and is based on the formulation of Kao and Michayluk, who attempted to define a medium for culturing protoplasts of the species *Vicia hajstana* at extremely low densities (25–50 pp mL^-1^) (Kao and Michayluk, 1975). To this end, the medium composition is more complex and contains, in addition to minerals, sucrose and glucose, several other sugars (fructose, ribose, xylose, mannose, rhamnose, cellobiose, sorbitol, and mannitol), organic acids (pyruvate, citrate, malate, and fumarate), 12 vitamins (from inositol to vitamin B_12_), casein hydrolysate, and coconut milk. Since its formulation, the medium has been simplified by several authors (Grzebelus et al., 2012; Kiełkowska and Adamus, 2014). Based on our statistical analysis, the changing of media based on Protocol 1 stimulated cell division and microcallus formation more efficiently, despite the complexity of the CPP medium, with abundant organic supplements used in Protocol 2. Both cauliflower cultivars showed statistically significantly higher percentages of dividing cells at a protoplast density of 10 x 10^4^ pp mL^-1^ when cultured according to Protocol 1 (**Supplementary Table 1**), which was in agreement with the subsequent differences in the number of microcallus colonies between the two protocols (**Figure 4B**). However, no statistically significant differences could be determined for the number of cauliflower microcalli due to the mean number of microcalli obtained under Protocol 2 of 0 in all biological replicates (95% CI 0.0–∞), which restricted the statistical analysis. The percentage of dividing cells after 5 or 15 days of culture, according to both protocols, was not an accurate indicator of the subsequent regeneration of microcalli because the latter depended on a small fraction of dividing cells that began to divide earlier and had divided more than twice, forming cell structures with more than 4 cells or even more than 8 cells until the 15^th^ day of culture (**Figure 5**). The cells in these structures continued to divide, and by day 20, they started to form macroscopically visible microcallus colonies, whereas a large number of smaller structures (2–4 cells) had obviously stopped dividing and most likely did not contribute to microcallus formation. Under Protocol 2, only the cabbage cultivar Reball formed microcalli (**Figure 4B**; **Table 2**) since its protoplasts were the only ones that had formed structures with more than 4 cells by day 15 (**Figure 5**). When cultured according to Protocol 1, all cultivars formed microcalli as they had formed structures with more than 4 cells by day 15; the cultivar All Year Round formed the highest number of microcallus colonies as it had also produced the highest number of structures with 8 or more cells by day 15. Thus, the larger structures were clearly composed of daughter cells of protoplasts that first entered reprogramming after immobilization and began with cell division. Other cells began to divide later, perhaps towards the end of the culture period in medium B under Protocol 1, and therefore could not divide successfully because medium C is less suitable for early cell division. As reported previously, the key to successful microcallus regeneration in the genus *Brassica* is the timely reduction of osmotic pressure, the change of the carbon source, and the modification of the hormone ratio (Kao and Michayluk, 1980; Kao and Seguin-Swartz, 1987; Yang et al., 1994; Li et al., 2021). Initially, a high ratio of auxins to cytokinins is required for cell wall synthesis and initiation of the first cell division, but at later stages, a lower ratio is required to promote further cell division. Also, initially, a high osmotic pressure is required to replace turgor, but this may impede nutrient uptake in the later stages. These modifications are included in the sequence of culture media based on Protocol 1, which was more suitable for cell division and microcallus formation of three of the four tested cultivars.

Evaluation of microcalli larger than 0.5 mm on day 30 proved to be a reliable estimation of the regeneration efficiency since on solid shoot regeneration medium, the smaller microcalli quickly turned brown and failed to develop further (**Figure 6**). A sufficient number of microcalli larger than 0.5 mm per alginate disc was needed for the successful further division of microcalli and differentiation into shoots. This was the main factor in shoot regeneration in the first experiment.

In our second experiment, we assessed the impact of adding 0.1 µM PSK to the culture medium during the initial 10 days to accelerate the onset of cell division. This acceleration is crucial for the subsequent regeneration of larger microcalli and the differentiation of shoots. Protoplasts from all four cultivars were cultured at an initial density of 10 x 10^4^ pp mL^-1^, which, according to previous studies, represents the optimal concentration for numerous plant species (Davey et al., 2010; Reed and Bargmann, 2021). Additionally, the lowest initial density of 2.5 x 10^4^ pp mL^-1^, which induced cell division in our preliminary experiment, was also employed.

This experiment revealed a beneficial effect of PSK as a mitogenic factor, stimulating early cell division. The intensity of its impact was strongly dependent on the genotype, with both cauliflower cultivars responding more robustly to the addition of PSK to the culture medium than the cabbage cultivars already until the 15^th^ day of cultivation (**Figure 7**, **8**; **Table 3**). In cauliflower, the addition of PSK led to a 2.2-fold (95% CI 1.0–4.7) to 5.3-fold (95% CI 3.1–9.1) higher frequency of cell division by day 15, with a more pronounced response observed at the higher protoplast density of 10 x 10^4^ pp mL^-1^ (**Supplementary Table 3**). Furthermore, the favorable effect of PSK on the number of microcalli larger than 0.5 mm was evident after 30 days of cultivation (**Figure 9B**; **Table 4**). Even at this stage, the impact of PSK was more pronounced in cauliflower, especially at a higher protoplast density, where it increased the number of the formed microcalli up to 37-fold (95% CI 1–1147) (**Supplementary Table 4**). Notably, in cabbage, there were no statistically significant differences in the number of microcalli with or without the addition of PSK to the culture medium after 30 days of cultivation. Our findings diverge from the previously reported stimulatory effects of PSK on six cabbage cultivars documented by Kiełkowska and Adamus (Kiełkowska and Adamus, 2019). This discrepancy could be attributed to the utilization of different genotypes in our experiments compared to theirs, as well as variations in the statistical analysis approaches employed. Nevertheless, in alignment with their study, our experiments confirmed an enhanced regeneration of shoots from calluses developed from cells treated with PSK during the early stages of development. Furthermore, supporting evidence from studies on carrot, a model species for *in vitro* cultures, revealed that the application of PSK during the initial protoplast culture led to a four-fold increase in the number of regenerated plants (Maćkowska et al., 2014).

Both preliminary experiments, Experiment 1 and Experiment 2, demonstrated that higher protoplast densities stimulate cell division and the formation of regenerated microcalli. In our third experiment, we successfully validated our hypothesis that PSK can compensate for the decline in protoplast viability following PEG-mediated transformation. Our results demonstrate that the viability of PEG-transformed protoplasts decreased to 32.5% after 2 days, whereas the viability of control (non-transformed) protoplasts remained at 81.9%. The low viability of treated protoplasts could thus be a major obstacle to the regeneration of plants from transformed protoplasts. These differences in the density of viable protoplasts translated into variations in the number of formed microcalli by day 30 (**Figure 10**; **Table 5**), which was statistically significantly higher in non-transformed compared to transformed protoplasts, showing 7.3-fold differences (95% CI 2.2–24.3), 18-fold differences (95% CI 2–164), and 17-fold differences (95% CI 2–155) at kanamycin concentrations of 10, 50, and 100 mg L^-1^, respectively (**Supplementary Table 5**). By adding PSK, we simulated a higher concentration, enabling the successful regeneration of transformed protoplasts even under strong selective pressure. The number of microcalli was 5.6-fold (95% CI 1.7–18.8), 44-fold (95% CI 5–389), and 38-fold (95% CI 4–337) higher when PSK was added to the medium (**Supplementary Table 5**).

All three of our experiments provide evidence that protoplast density influences the success of cell division and the formation of microcalli, prerequisites for the subsequent development of larger callus clusters and their differentiation into shoots. We could also confirm that PSK supplementation facilitates the regeneration of calli and shoots even at low densities of immobilized viable protoplasts.

## 5 Conclusions

The scientific literature features numerous reports on the role of PSK in plant growth and development *in vivo*; however, its implications in plant biotechnology and breeding are understudied or, at the very least, underreported. To the best of our knowledge, our study represents the first exploration into the stimulatory impact of PSK on the regeneration of cauliflower protoplasts or any other cauliflower cell type or tissue. Hitherto, within the *Brassica* genus, PSK has exclusively demonstrated its stimulatory effect during the regeneration of cabbage protoplasts (Kiełkowska and Adamus, 2019) and oilseed rape microspore embryogenesis (Mestinšek Mubi et al., 2024). Notably, the application of PSK has not been investigated in PEG-treated protoplasts of any plant species. Interestingly, in the context of *Agrobacterium*-mediated genetic transformation of carrot hypocotyl explants, the addition of PSK to selective media substantially increased the recovery of transformed calli from 7% to 39% (Matsubayashi et al., 2004). Our report, revealing a remarkable up to 44-fold increase in the number of microcalli formed from PEG-mediated transformed protoplasts due to PSK supplementation, highlights the potent stimulatory effect of PSK. This finding opens new avenues for future investigations into the regeneration of transformed protoplasts, capitalizing on the robust impact of PSK demonstrated in our study.

## Data availability statement

The original contributions presented in the study are included in the article/**Supplementary Material**. Further inquiries can be directed to the corresponding author.

## Author contributions

VV: Conceptualization, Investigation, Methodology, Writing – original draft. DK: Formal analysis, Software, Visualization, Writing – review & editing. JM: Conceptualization, Data curation, Formal analysis, Funding acquisition, Investigation, Methodology, Project administration, Resources, Software, Supervision, Validation, Visualization, Writing – original draft, Writing – review & editing.
