# Supplementary material for: Phytosulfokine alpha enhances regeneration of transformed and untransformed protoplasts of Brassica oleracea
Source: Front Plant Sci. 2024 Mar 27;15:1379618. doi: 10.3389/fpls.2024.1379618 (PMC11004253; doi:10.3389/fpls.2024.1379618)
Supplement: Supplementary file 1 [file Table_1.pdf]

Phytosulfokine alpha enhances regeneration of transformed and untransformed protoplasts of *Brassica oleracea*

Valentin Vogrinčič[1], Damijana Kastelec[1] and Jana Murovec[1*]

Biotechnical Faculty, University of Ljubljana, 1000 Ljubljana, Slovenia

[*] Corresponding author: jana.murovec@bf.uni-lj.si; Tel.: +386 1 320 3265; ORCID identifier 0000-0002-6099-4057

Exp 1: Cell divisions

Supplementary Table 1: Estimated pairwise ratios (fold changes) of mean percentages of dividing cells showing a statistically significant difference from the value of 1, based on the Poisson regression model comparing different levels of the three factors analysed in Experiment 1: four *B. oleracea* cultivars, two culture protocols, and three protoplast densities. The ratios represent the percent changes in dividing cells after 15 days of culture.

**Contrast: Protocols**	**Density (x 10^4 pp ml^{-1})**	**Cultivar**	**Ratio**	**SE**	**95% CI**
Protocol 1 / Protocol 2	10	AYR[1]	2.31	0.51	1.49-3.57
Protocol 1 / Protocol 2	10	Erfurt	1.85	0.45	1.15-2.98
Protocol 1 / Protocol 2	5	Reball	2.25	0.48	1.48-3.41
Protocol 1 / Protocol 2	2.5	Reball	2.20	0.84	1.04-4.65
**Contrast: Density**	**Protocol**	**Cultivar**	**Ratio**	**SE**	**95% CI**
10 / 2.5	Protocol 1	AYR	3.53	0.92	1.92-6.49
10 / 2.5	Protocol 1	Erfurt	3.20	0.95	1.60-6.40
10 / 2.5	Protocol 1	Huzaro	4.22	1.11	2.28-7.81
10 / 2.5	Protocol 1	Reball	3.55	0.86	2.01-6.24
10 / 2.5	Protocol 2	Huzaro	1.74	0.39	1.03-2.95
10 / 2.5	Protocol 2	Reball	6.00	2.05	2.70-13.36
10 / 5	Protocol 1	AYR	2.09	0.45	1.26-3.47
10 / 5	Protocol 1	Erfurt	2.09	0.53	1.15-3.78
10 / 5	Protocol 1	Huzaro	2.05	0.41	1.28-3.29
10 / 5	Protocol 2	Reball	1.88	0.41	1.12-3.13
5 / 2.5	Protocol 1	Huzaro	2.06	0.59	1.05-4.03
5 / 2.5	Protocol 1	Reball	3.27	0.80	1.85-5.79
5 / 2.5	Protocol 2	Reball	3.20	1.16	1.37-7.48
**Contrast: Cultivar**	**Protocol**	**Density (x 10^4 pp ml^{-1})**	**Ratio**	**SE**	**95% CI**
AYR / Huzaro	Protocol 2	5	0.50	0.13	0.25-0.99
AYR / Huzaro	Protocol 2	10	0.54	0.12	0.30-0.97
Erfurt / Huzaro	Protocol 2	5	0.33	0.10	0.15-0.74
Erfurt / Huzaro	Protocol 2	10	0.48	0.11	0.26-0.89
AYR / Reball	Protocol 1	5	0.44	0.09	0.26-0.77
AYR / Reball	Protocol 2	10	0.48	0.11	0.27-0.86
Erfurt / Reball	Protocol 1	5	0.32	0.08	0.17-0.59
Erfurt / Reball	Protocol 1	10	0.62	0.11	0.38-0.99
Erfurt / Reball	Protocol 2	10	0.43	0.10	0.24-0.79
Huzaro / Reball	Protocol 1	5	0.51	0.10	0.31-0.86
Huzaro / Reball	Protocol 2	2.5	3.10	1.13	1.22-7.89

[1] AYR – All Year Round

Exp 1: Microcalli formation

Supplementary Table 2: Estimated pairwise ratios (fold changes) of mean numbers of microcalli showing a statistically significant difference from the value of 1, based on the negative binomial regression model comparing the different levels of the three factors analysed in Experiment 1: four *B. oleracea* cultivars, two culture protocols, and three protoplast densities. The ratios represent the changes in the number of microcalli after 30 days of culture.

**Contrast: Protocols**	**Density (x 10^4 pp ml^{-1})**	**Cultivar**	**Ratio**	**SE**	**95% CI**
Protocol 1 / Protocol 2	10	Huzaro	38.00	39.00	5.07-284.7
Protocol 1 / Protocol 2	10	Reball	0.34	0.09	0.20-0.58
Protocol 1 / Protocol 2	5	Reball	3.75	1.62	1.60-8.77
**Contrast: Density**	**Protocol**	**Cultivar**	**Ratio**	**SE**	**95% CI**
10 / 2.5	Protocol 1	AYR	8.22	2.49	4.04-16.72
10 / 2.5	Protocol 1	Erfurt	5.17	2.47	1.69-15.83
10 / 2.5	Protocol 1	Huzaro	12.67	7.90	2.94-54.65
10 / 2.5	Protocol 1	Reball	10.33	6.49	2.37-45.08
10 / 5	Protocol 1	AYR	1.81	0.40	1.08-3.02
10 / 5	Protocol 1	Erfurt	3.44	1.43	1.30-9.13
10 / 5	Protocol 1	Huzaro	2.53	0.89	1.12-5.75
10 / 5	Protocol 2	Reball	11.25	4.58	4.34-29.19
5 / 2.5	Protocol 1	AYR	4.56	1.42	2.19-9.46
5 / 2.5	Protocol 1	Huzaro	5.00	3.28	1.08-23.23
5 / 2.5	Protocol 1	Reball	10.00	6.29	2.29-43.71
**Contrast: Cultivar**	**Protocol**	**Density (x 10^4 pp ml^{-1})**	**Ratio**	**SE**	**95% CI**
AYR / Huzaro	Protocol 1	2.5	6.00	3.88	1.14-31.61
AYR / Huzaro	Protocol 1	5	5.47	1.80	2.35-12.73
AYR / Huzaro	Protocol 1	10	3.89	0.97	2.05-7.40
AYR / Reball	Protocol 1	2.5	6.00	3.88	1.14-31.61
AYR / Reball	Protocol 1	5	2.73	0.75	1.35-5.52
AYR / Reball	Protocol 1	10	4.77	1.25	2.44-9.35
AYR / Erfurt	Protocol 1	5	9.11	3.56	3.34-24.87
AYR / Erfurt	Protocol 1	10	4.77	1.25	2.44-9.35
Erfurt / Reball	Protocol 1	5	0.30	0.13	0.10-0.88
Huzaro / Reball	Protocol 2	10	0.01	0.10	0.00-0.15

Exp 2: Mitogenic activity of PSK

Supplementary Table 3: Estimated pairwise ratios (fold changes) of the mean percentage of dividing cells showing a statistically significant difference from the value of 1, based on the Poisson regression model comparing different levels of the three factors analysed in Experiment 2: four *B. oleracea* cultivars, two protoplast densities, and supplementation of liquid medium with 0.1 µM PSK. The ratios represent the percentage changes in dividing cells after 15 days of culture.

**Contrast: Supplementation**	**Density (x 10^4 pp ml^{-1})**	**Cultivar**	**Ratio**	**SE**	**95% CI**
PSK+ / PSK-	10	AYR	3.77	0.98	2.27-6.27
PSK+ / PSK-	2.5	AYR	2.20	0.85	1.03-4.69
PSK+ / PSK-	10	Erfurt	5.32	1.47	3.10-9.14
PSK+ / PSK-	2.5	Erfurt	2.81	1.03	1.37-5.75
**Contrast: Density**	**Protocol**	**Cultivar**	**Ratio**	**SE**	**95% CI**
10 / 2.5	PSK+	AYR	3.29	0.81	2.03-5.33
10 / 2.5	PSK+	Erfurt	2.92	0.63	1.91-4.46
10 / 2.5	PSK+	Huzaro	2.63	0.87	1.37-5.04
10 / 2.5	PSK-	Huzaro	3.54	1.26	1.76-7.10
10 / 2.5	PSK+	Reball	3.41	1.15	1.76-6.62
10 / 2.5	PSK-	Reball	3.20	1.07	1.66-6.17
**Contrast: Cultivar**	**Supplementation**	**Density (x 10^4 pp ml^{-1})**	**Ratio**	**SE**	**95% CI**
AYR / Huzaro	PSK+	10	2.15	0.46	1.25-3.71
AYR / Reball	PSK+	10	1.83	0.37	1.09-3.06
Erfurt / Huzaro	PSK+	10	2.54	0.52	1.49-4.31
Erfurt / Reball	PSK+	10	2.15	0.42	1.31-3.55
Erfurt / Huzaro	PSK-	10	0.43	0.13	0.20-0.95
Erfurt / Reball	PSK-	10	0.42	0.13	0.19-0.90
Erfurt / Reball	PSK+	2.5	2.52	0.88	1.02-6.20

Exp 2: Effect of PSK on microcalli formation

Supplementary Table 4: Estimated pairwise ratios (fold changes) of mean numbers of microcalli showing a statistically significant difference from the value of 1, based on the negative binomial regression model comparing the different levels of the three factors analysed in Experiment 2: four *B. oleracea* cultivars, two protoplast, densities and supplementation of liquid medium with 0.1 µM PSK. The ratios show fold changes in the number of microcalli after 30 days of culture.

**Contrast: Supplementation**	**Density (x 10^4 pp ml^{-1})**	**Cultivar**	**Ratio**	**SE**	**95% CI**
PSK+ / PSK-	10	AYR	1.75	0.42	1.09-2.81
PSK+ / PSK-	2.5	AYR	7.50	6.90	1.23-45.56
PSK+ / PSK-	10	Erfurt	18.62	11.69	5.44-63.72
PSK+ / PSK-	2.5	Erfurt	37.00	64.84	1.19-1147.70

**Contrast: Density**	**Protocol**	**Cultivar**	**Ratio**	**SE**	**95% CI**
10 / 2.5	PSK+	AYR	4.67	1.62	2.36-9.23
10 / 2.5	PSK-	AYR	20.00	17.72	3.52-113.54
10 / 2.5	PSK+	Erfurt	4.03	1.28	2.16-7.50
10 / 2.5	PSK+	Huzaro	5.00	3.16	1.45-17.24
10 / 2.5	PSK-	Huzaro	4.50	3.04	1.20-16.93
10 / 2.5	PSK+	Reball	13.67	14.13	1.80-103.75
10 / 2.5	PSK-	Reball	14.67	15.13	1.94-110.82

**Contrast: Cultivar**	**Supplementation**	**Density (x 10^4 pp ml^{-1})**	**Ratio**	**SE**	**95% CI**
AYR / Huzaro	PSK+	10	3.11	0.92	1.45-6.66
AYR / Reball	PSK+	10	3.41	1.05	1.55-7.52
Erfurt / Huzaro	PSK+	10	3.31	0.97	1.56-7.05
Erfurt / Reball	PSK+	10	3.63	1.11	1.66-7.96
AYR / Erfurt	PSK-	10	10	6.41	1.93-51.93

Exp 3: Effect of PSK on microcalli formation from transformed protoplasts

Supplementary Table 5: Estimated pairwise ratios (fold changes) of mean numbers of microcalli showing a statistically significant difference from the value of 1, based on the Poisson regression model comparing different levels of the three factors analysed in Experiment 3: transformation of protoplasts, supplementation of liquid medium with 0.1 µM PSK, and kanamycin at three concentrations. The ratios represent fold changes in the number of microcalli after 30 days of culture.

Contrast: Transformation	Supplementation	Kanamycin (mg l^{-1})	Ratio	SE	95% CI
Untransformed / Transformed	PSK-	100	17.00	19.19	1.86-155.27
Untransformed / Transformed	PSK-	50	18.00	20.29	1.98-163.94
Untransformed / Transformed	PSK-	10	7.29	4.48	2.19-24.29

Contrast: Supplementation	Transformation	Kanamycin (mg l^{-1})	Ratio	SE	95% CI
PSK+ / PSK-	Transformed	100	38.00	42.34	4.28-337.36
PSK+ / PSK-	Transformed	50	44.00	48.95	4.97-389.39
PSK+ / PSK-	Transformed	10	5.57	3.45	1.66-18.75
